# Phytochemical Analysis Using UPLC-MS/MS Combined with Network Pharmacology Methods to Explore the Biomarkers for the Quality Control of Lingguizhugan Decoction

**DOI:** 10.1155/2021/7849032

**Published:** 2021-12-22

**Authors:** Baolin Li, Shuaishuai Fan, Jingnan Hu, Yongben Ma, Yu Feng, Fengxia Wang, Xinguo Wang, Liying Niu

**Affiliations:** ^1^College of Integrative Medicine, Hebei University of Chinese Medicine, Shijiazhuang 050091, China; ^2^Hebei Traditional Chinese Medicine Formula Granule Engineering & Technology Innovate Center, Shijiazhuang 050091, China; ^3^Quality Evaluation & Standardization Hebei Province Engineering Research Center of Traditional Chinese Medicine, Shijiazhuang 050091, China

## Abstract

As a classic TCM prescription, LGZG has been widely used in clinical prevention and treatment of heart failure, nonalcoholic fatty liver, and hyperlipidemia. However, there are few studies on chemical components in recent years, and the basis of quality evaluation is not sufficient. This study was to find the active ingredients of the Lingguizhugan decoction using UPLC-MS/MS and network pharmacology. By comparing the retention time and MS dates of the reference and self-building database, the cleavage rules of chemical composition whose mass errors are less than 1 ppm(FL less than 3 ppm) are analyzed. On this basis, a network pharmacology method was used to find biomarkers for quantitative analysis. The results show that 149 compounds were preliminaries identified or inferred, including 63 flavonoids, 30 triterpenes, 22 phenylpropanoids, 13 organic acids, 6 lactones, 5 alkaloids, 4 anthraquinones, and 6 other compounds. According to the network pharmacology results, 20 chemical constituents were selected as the biomarkers, which were determined simultaneously for the first time, including poricoic acid A, poricoic acid B, glycyrrhizic acid, glycyrrhetinic acid, liquiritin, isoliquiritin, liquiritigenin, isoliquiritin apioside, cinnamic acid, caffeic acid, neochlorogenic acid, chlorogenic acid, cryptochlorogenic acid, isochlorogenic acid A, B, and C, atractylenolide I, II, and III, and coumarin. The methodological results show that the linearity, stability, precision, repeatability, and recovery of the method are satisfactory. Therefore, a comprehensive quality assessment system for LGZG was established on the basis of a systematic study of chemical substances and network pharmacology, which provided an important reference for the foundation of pharmacological action and its mechanics.

## 1. Introduction

Lingguizhugan decoction (LGZG), coming from Zhang Zhongjing's Synopsis of Preions of the Golden Chamber, has already been listed as the first batch of “The Catalogue of ancient classical prescription” by the National Administration of Traditional Chinese Medicine (TCM) in 2018. LGZG is prepared by four different herbal medicines, namely Fuling (the dry sclerotia of *Poria cocos* (Schw.), FL), Guizhi (the dry twigs of *Cinnamomum cassia* Presl, GZ), Baizhu (the dry rhizome of *Atractylodes macrocephala* Koidz, BZ), and Gancao (the dry root and rhizome of *Glycyrrhiza uralensis* Fisch., *Glycyrrhiza inflata* Bat. Or *Glycyrrhiza glabra* L., GC). Modern pharmacological studies have revealed that LGZG has a variety of biological and pharmacological properties, such as the regulation of cardio-cerebrovascular function [1], regulation of lipid metabolism and blood circulation [2], and anti-inflammation effects [3]. At present, LGZG has been widely used in clinics for the prevention and treatment of heart failure [4], acute myocardial infarction [5], nonalcoholic fatty liver [6], obesity [7], hyperlipidemia [8], gastrointestinal function regulation [9], and many other diseases [10, 11]. However, combined with the present stage of research, most of the studies on LGZG have been restricted to the main research index of licorice-related components, while the related components in other herbal medicines are rarely involved, or the overall quality evaluation indexes are not comprehensive, or the range of mass error is excessive, which results in a limited accuracy [12, 13]. Considering that licorice is only used as a guide medicine in the prescription, the comprehensiveness and objectivity of the method cannot be guaranteed.

As we all know, compound prescriptions of TCM are related to many different herbal medicines, which contain a variety of chemical components and more complex interactions. Generally speaking, after oral administration, the chemical components of the drugs can produce specific synergism or antagonism through blood circulation in multiple organs and targets of the whole body. Its prototype components or metabolites play a corresponding therapeutic role through specific pathways, such as participating in the metabolic regulation, or regulating the homeostasis of microorganisms, and finally causing the overall level of metabolites to change to the normal level. Therefore, it is particularly necessary to establish a comprehensive and systematic research for the chemical constituents of LGZG.

Thus, our study established a quality evaluation system for LGZG using both UPLC-MS/MS and network pharmacology method, which has important guiding significance to help us clarify the pharmacodynamic material basis and its mechanism.

## 2. Materials and Methods

### 2.1. Materials and Reagents

The abovementioned Chinese herbal medicines were purchased from the following places of origin: FL (batch number: YF18081902, source: Yunnan China; batch number: YF18081914, source: Anhui China; batch number: FL180315, source: Shanxi China), GZ (batch number: YF18083004, source: Guangxi-Botang China; batch number: GZ180321, source: Guangdong China; batch number: 1710203, source: Guangxi-Guilin China), BZ (batch number: YF18070702, source: Zhejiang China; batch number: YF18070603, source: Hebei China; batch number: YF18070802, source: Anhui China), and GC(batch number: YF18063004, source: Neimeng China; batch number: YF18062905, source: Xinjiang China; batch number: YF18062907, source: Gansu China) were provided by Jinnuokang Co., Ltd. (Shijiazhuang, China), which followed identification by Professor Baohui Sun (Hebei Provincial Drug Inspection Institute, Shijiazhuang, China). The voucher specimens have been deposited in the herbarium of the Hebei University of Chinese Medicine.

The purities of all reference standards were higher than 93%, containing cinnamic acid, cinnamaldehyde, atractylenolide I, atractylenolide II, atractylenolide III, liquiritin, glycyrrhetinic acid, caffeic acid, chlorogenic acid, neochlorogenic acid, and cryptochlorogenic acid attained from the National Institutes for Food and Drug Control (Beijing, China); poricoic acid A, poricoic acid B, polyporenic acid C, isochlorogenic acid A, isochlorogenic acid B, and isochlorogenic acid C were purchased from the Chengdu Desite Bio-Technology Co., Ltd. (Chengdu, China); isoliquiritin apioside and coumarin were obtained from Shanghai Yuanye Bio-Technology Co., Ltd. (Shanghai, China); isoliquiritin, dehydrotumulosic acid, pachymic acid, 4-hydroxybenzoic acid, and neoisoliquiritin were purchased from Chengdu Pusi Bio-Technology Co., Ltd. (Chengdu, China); glycyrrhizic acid, liquiritigenin, and rutin were obtained from Chengdu Mansite Bio-Technology Co., Ltd. (Chengdu, China); and licochalcone A was purchased from Chengdu Pufei De Bio-Technology Co., Ltd. (Chengdu, China).

Methanol of the HPLC grade was obtained from Fisher Scientific (Fair Lawn, NJ, USA). Acetonitrile of the HPLC grade, together with formic acid of the LC-MS grade, was purchased from Merck (Darmstadt, Germany). Purified water was purchased from Watsons Corporation (Guangzhou, China). The vacuum freeze-dryer was purchased from Beijing Boyikang Experimental Instrument (Beijing, China). All other chemicals and reagents used were of HPLC grade.

### 2.2. Standards and Sample Preparation

#### 2.2.1. Sample Preparation

Following the ancient prescription, herbal medicines, including FL 55.2 g, GZ 41.4 g, BZ 41.4 g, and GC 27.6 g, were taken together with 1200 mL purified water. Until the extracted solution remained 600 mL, it was filtered through a 200-mesh sieve and freeze-dried. For details of the origin of 18 batches of sample medicinal materials, see Table 1

The sample of LGZG powder(1.0 g) was accurately weighed, dissolved in 70% methanol-water (25 mL), and subjected to ultrasonic extraction for 15 min. The solution was weighed again and after the loss in weight was made up with methanol, the obtained solution was then subjected to centrifugation at 4000 rpm for 10 min, and the supernatant was filtered through a 0.22 µm nylon membrane (Millipore, USA) prior to analysis. All solutions are stored at 4°C before use.

#### 2.2.2. Preparation of Reference Solutions

The reference substance, with the precise weight, was dissolved in HPLC Grade methanol separately. A final mixed standard solution was prepared from all the solutions with the concentrations of poricoic acid A 0.02100 *μ*g/mL, poricoic acid B 0.02150 *μ*g/mL, glycyrrhizic acid 28.29 *μ*g/mL, glycyrrhetinic acid 0.03168 *μ*g/mL, liquiritin 32.64 *μ*g/mL, isoliquiritin 4.212 *μ*g/mL, liquiritigenin 12.33 *μ*g/mL, isoliquiritin apioside 5.362 *μ*g/mL, cinnamic acid 7.096 *μ*g/mL, caffeic acid 0.1697 *μ*g/mL, neochlorogenic acid 1.655 *μ*g/mL, chlorogenic acid 1.615 *μ*g/mL, cryptochlorogenic acid 0.5528 *μ*g/mL, isochlorogenic acid A 0.3894 *μ*g/mL, isochlorogenic acid B 0.2430 *μ*g/mL, isochlorogenic acid C 0.3353 *μ*g/mL, atractylenolide I 0.6000 *μ*g/mL, atractylenolide II 0.3300 *μ*g/mL, atractylenolide III 0.9400 *μ*g/mL and coumarin 2.736 *μ*g/mL. All the standard solutions were stored at 4°C and filtered through a 0.22 µm nylon membranes (Millipore, USA) before analysis.

### 2.3. Chromatographic and Mass Spectral Conditions

#### 2.3.1. UPLC-Q-TOF-MS/MS Analysis

UPLC-Q-TOF-MS/MS analysis was completed with an Acquity TM UPLC system (Waters, Milford, MA, USA), which was equipped with a quaternary solvent manager, an automatic sample manager-FTN, and a PDA e*λ* detector. The samples were separated on a Waters ACQUITY UPLC BEH C18(100 mm × 2.1 mm, 1.7 *μ*m) maintained at 35°C, using a mixed mobile phase consisted of 0.1% formic acid in pure water (A) and acetonitrile (B). A gradient elution program was as follows: 0–3 min, 5% B; 3–20 min, 5%–30% B; 20–25 min, 30% B; 25–50 min, 30%–80% B; 50–55 min, 80% B; and 55.1–60 min, 5% B. The flow rate and the injection volume were set at 0.3 mL/min and 2 *μ*L, respectively.

The mass analysis was implemented by a Triple TOFTM 6600 + (AB SCIEX, Foster City, CA, USA), which is equipped with an electrospray ionization (ESI) source. The MS analysis was performed in both positive ionization mode and negative ionization mode by full scan mode. The optimized parameters were as follows: ion spray voltage (ISV), 5.5 kV (ESI+) or −4.5 kV (ESI-); ion source temperature (TEM), 550°C; ion source gas 1 (GS1), 55 psi; ion source gas 2 (GS2), 55 psi; curtain gas (CUR), 35 psi, scan range of TOF-MS, m/*z* 100–1500 Da; and scan range of product ion, m/*z* 100–1500 Da. IDA-MS/MS conditions were as follows: accumulation time was 0.05 s; high sensitivity mode was set, excluding isotopes were within 4 Da; declustering potential (DP) was 80V (ESI+) or −80V (ESI-); collision energy (CE) was 10 eV (MS mode), and 40 and 80 eV (MS2 mode); and collision energy spread (CES) was 20 eV. Moreover, in order to ensure accurate mass measurements during the MS experiments, the instrument performed mass accuracy calibration through the CDS system before each experiment. During the experiment, the mass accuracy was calibrated for every three samples. The data acquisition and processing will be analyzed by SCIEX OS-Q 2.0 Software (AB SCIEX, Foster City, CA, USA) and Peak View® 2.2 Software (AB SCIEX, Foster City, CA, USA).

#### 2.3.2. UPLC-QQQ-MS/MS Analysis

UPLC-QQQ-MS/MS analysis was performed on the LC-30A UPLC system(Shimadzu, Kyoto, Japan), including DGU-30A3 type online vacuum degasser, LC-30AD-type binary pump, SIL-30AC-type automatic sampler, and CTO-30A-type column incubator. The experimental conditions were as follows: Shim-pack GIST C18(100 mm × 2.1 mm, 2 *μ*m) chromatographic column; column temperature, 35°C; flow rate, 0.3 mL/min; and injection volume, 2 *μ*L. The mobile phase was selected to be water containing 0.1% formic acid (A) and acetonitrile (B) with the following gradient elution programme: 0–17 min, 5–17% B; 17–31 min, 17–75% A; 31–32 min, 75% A; and 32.1–35 min, 5% A. In terms of mass spectrometry, we chose QTRAP 4500 (AB SCIEX, Foster City, CA, USA) coupled with an electrospray ionization (ESI) source. The analytical method adopts the MRM mode, with the positive and negative ion switching detection method. The ion spray voltage (ISV) was 5.5 kV (ESI+) or −4.5 kV (ESI-); ion source temperature, (TEM) 550°C; ion source gas 1 (GS1), 55 psi; ion source gas 2 (GS2), 55 psi; and curtain gas (CUR), 35 psi.

### 2.4. Validation of the Quantitative Method

#### 2.4.1. Calibration Curve, LOD, and LOQ

The mixed reference substance solution was diluted step by step to form a mixed reference substance solution with six concentration gradients. According to the condition of item 2.3.2, the extracted ion current chromatogram of each reference substance was obtained. With the concentration of each reference substance (X) and peak area (Y), the standard curves were drawn and linear regressions were carried out. The mixed reference solution was diluted step by step and determined until the quantification limit (LOQ) and detection limit (LOD) were determined when the signal-to-noise ratio (S/N) was 10 and 3, respectively.

#### 2.4.2. Precision, Repeatability, Stability, and Recovery

In order to investigate the precision, the mixed reference solution and sample solution were injected continuously for 6 times, the peak areas were recorded, and the RSD values were calculated. In the repetitive investigation, 6 samples of the same batch were taken and prepared in parallel. Through the sequential injection analysis of the samples, the peak area and the RSD value were calculated. According to the stability test, the solution of the same batch of samples was injected and analyzed at 0, 4, 8, 12, 18, 24, 36, and 48h, respectively. The recovery rates were investigated by adding high, medium, and low levels of reference solution (150, 100, and 50% of the known amount, respectively) to the same batch of samples, with 3 parallel samples at each level. Recovery (%) = (detected value – original value)/added amount × 100%.

### 2.5. Strategy

Through searching and sorting out the previous literature, the chemical composition information database of four kinds of TCM in LGZG was established, including compound name, molecular formula, exact molecular weight, structural formula, and other essential information. For compounds with reference substances, it was confirmed by comparing the chromatographic retention time and mass spectrometry data. For unknown compounds, the OS software was used to compare the self-built composition database, TCM MS/MS database (supplied by AB SCIEX) and online Chemspider database. The screening conditions were that the quality deviation was less than 1 ppm (FL less than 3 ppm). The fragmentation pattern of representative components was analyzed by the MS/MS spectrogram of the compound.

According to the results of chemical composition research, a network pharmacology research was carried out to screen biomarkers, which were taken as the references to carry out the quality evaluation research. On this basis, the study established a quantitative study with high accuracy and good stability. Combined with the method of statistical analysis, the differences between different areas of herbal medicines were analyzed, which will provide a theoretical basis for the quality evaluation of TCM.

## 3. Results and Discussion

### 3.1. Identification of the Chemical Components in LGZG by UPLC-ESI-Q-TOF-MS

**Figure 1:**
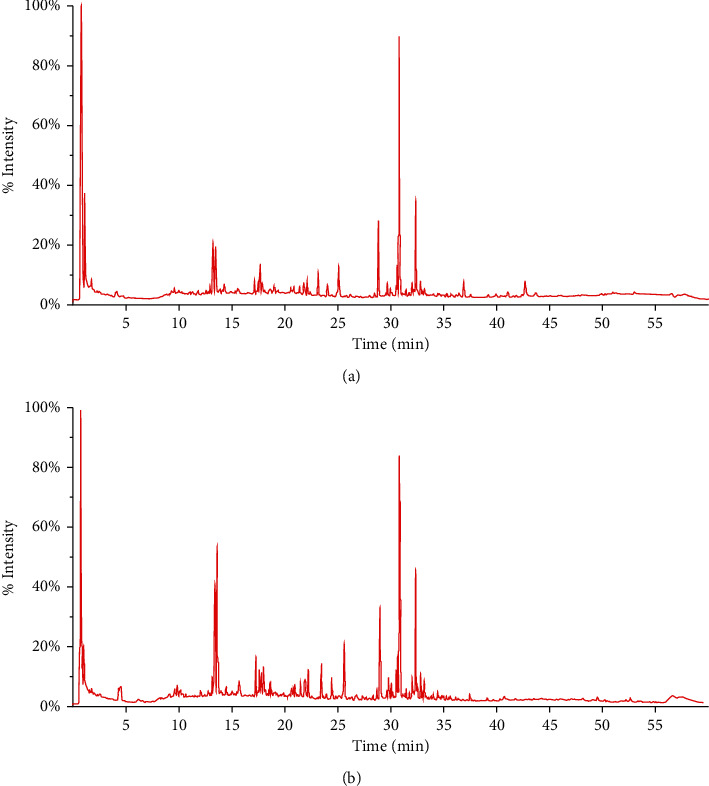
The total ion chromatograms of LGZC (a) under the positive ion mode and (b) under the negative ion mode.

By introducing the UPLC-ESI-Q-TOF-MS analysis method based on DBS-IDA data acquisition technology, the spectrum signals were 45,205 and 43,188 measured in positive mode and negative ion detection mode, respectively. Combined with reference substance comparison, literature research, and MS/MS information, a total of 149 chemical constituents in LGZG were identified, including 63 of flavonoids, 30 of triterpenes, 22 of phenylpropanoids, 13 of organic acids, 6 of lactones, 5 of alkaloids, 4 of anthraquinones, and 6 of other compounds. The typical total ion chromatograms of positive and negative ion modes are shown in Figure 1. All the compounds and related information are shown in Tab. S1.

#### 3.1.1. Identification of Flavonoids

As the most abundant compounds in nature, flavonoids are not only the main chemical components of many kinds of TCM, but also the most important therapeutic active components. In this study, a total of 63 flavonoids were identified or preliminarily inferred, mainly from GZ and GC. These components can be further divided into flavonoid aglycones, O-glycosyl flavonoids, and C-glycosyl flavonoids.

**Figure 2:**
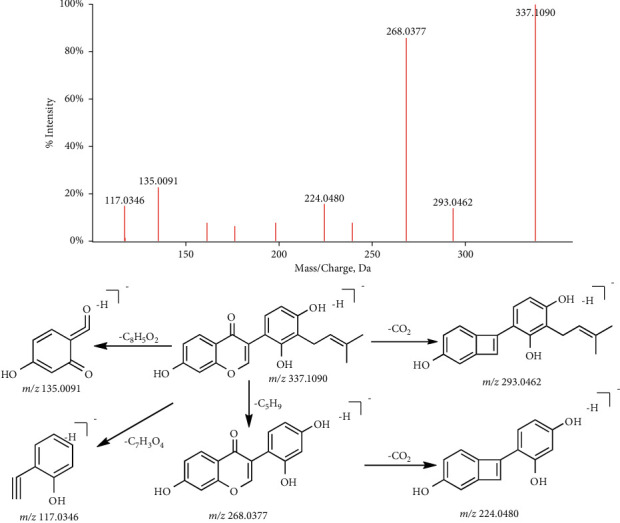
The fragmentation pathway of eurycarpin A.

*(1) Flavonoid Aglycones*. The main cracking mode of flavonoids is the Retro-Diels–Alde (RDA) fragmentation mechanism, which can also eliminate small neutral molecular fragments, such as CH_3_ (m/*z* 15.0240 Da), O (m/*z* 15.9949 Da), H_2_O (m/*z* 18.0106 Da), CO (m/*z* 27.9949 Da), CO_2_ (m/*z* 43.9898 Da), and other substituents on benzene ring [14–16]. Take isoflavones Eurycarpin A (C_20_H_18_O_5_, [M-H]^−^, P103) as an example, which is observed at m/*z* 337.1090. Firstly, the fragment ion m/*z* 293.0462 is produced by losing CO_2_. Meanwhile, the molecule can remove the isopentenyl group (C_5_H_9_) from the benzene ring to form the m/*z* 268.0377 fragment ion (m/*z* -69), which can continue to remove –CO_2_ to form m/*z* 224.0480 fragment ion. Finally, on the basis of 368 fragments, m/*z* 135.0091 and m/*z* 117.0346 fragments are produced through the RDA fragmentation mechanism. The fragmentation pathway of Eurycarpin A is shown in Figure 2.

As a branch of flavonoids, isoflavanes do not contain CO in the C_3_ ring, which cannot form a conjugated system.

**Figure 3:**
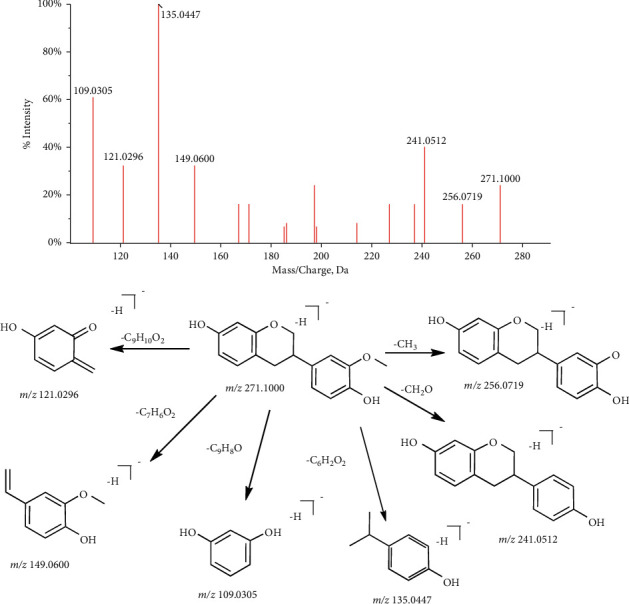
The fragmentation pathway of 7,4′-dihydroxy-3′-methoxyisoflavan.

This leads to its unstable structure and easy fracture at the C_3_ ring. We explain the fragmentation mechanism of isoflavane through compounds 7,4′-dihydroxy-3′-methoxy-isoflavan (C_16_H_16_O_4_, [M-H]^−^, P87). The component exhibited a parent ion at m/*z* 271.1000, which yielded daughter ions at m/*z* 256.0719 and 241.0512 by losing a CH_3_ and CH_2_O group, respectively. Besides, components were cleaved by RDA reaction to form m/*z* 121.0296 and 149.0600. The most important thing is that the C–C and C–O bonds at C_3_ can be broken, so that the molecule is divided into two new fragment ions m/*z* 109.0305 and 135.0447. The fragmentation pathway of 7,4′-dihydroxy- 3′-methoxyisoflavan was shown in Figure 3.

According to the rules mentioned above, a total of 46 flavonoids were identified or inferred, including 13 isoflavones (labeled as P50, 84, 85, 103, 110, 115, 119, 120, 123, 132, 135, 140, 141), 8 dihydroflavonoids(labeled as P41, 67, 81, 100, 101, 118, 125, 131), 8 chalcone (labeled as P58, 72, 73, 74, 83, 97, 109, 112), 5 flavonoids(labeled as P49, 60, 65, 114, 124), 3 isoflavones(labeled as P87, 99, 126), 2 flavonols (labeled as P51, 76), 2 dihydroflavonols(labeled as P21, 39), 2 procyanidins(labeled as P20, 28), 1 xanthones(labeled as P47), 1 isoflavene (labeled as P93), and 1 2-(2-phenylethyl) chromone(labeled as P69).

*(2) O-Glycosyl Flavonoids.* Natural flavonoids mainly exist in plants in the form of glycosides combined with glycosyl groups (monosaccharides, disaccharides, trisaccharides, polysaccharides, etc.). According to the glycoside bond atoms connected to the glycosyl groups, natural flavonoids are divided into O-glycosyl flavonoids and C-glycosyl flavonoids.

For O-flavonoid glycosides, the glycosyl connection position is related to the parent nuclear structure of flavonoids. In terms of flavonoids, dihydroflavonoids and isoflavones, most of them form monosaccharides on 7-OH. While flavonols and dihydroflavonol glycosides form monosaccharides on 3-OH, 7-OH, 3′-OH and 4′-OH, or disaccharides on 3-OH and 7-OH, 3-OH and 4′-OH or 7-OH and 4′-OH.

**Figure 4:**
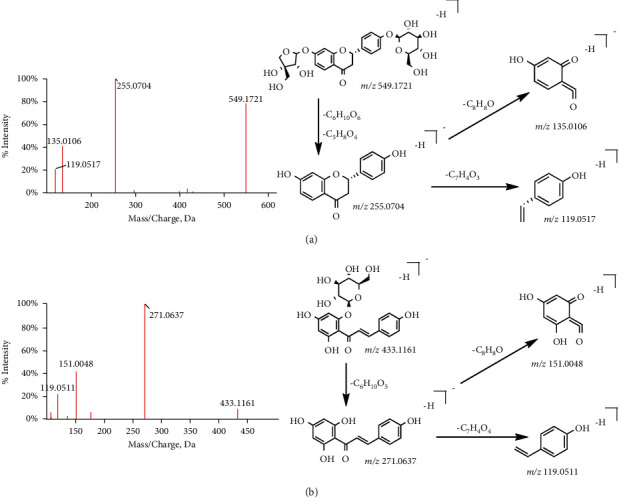
The fragmentation pathway of O-glycosyl flavonoid. The fragmentation pathway of (a) liquiritin apioside and (b) isosalipurposide.

Dihydroflavone O-glycoside liquiritin apioside (C_26_H_30_O_13_, [M-H]^−^, P45) and chalcone O-glycoside isosalipurposide (C_21_H_22_O_10_, [M-H]^−^, P59) are used as standards for explanation. The two compounds are first removed from the glycosyl group: liquiritin apioside removes two molecules of glycosyl (C_5_H_8_O_4_, m/*z* 132.0423; C_6_H_10_O_5_, m/*z* 162.0528); and isosalipurposide loses a molecule(C_6_H_10_O_5_). On this basis, new fragment ions are further formed by RDA cleavage (liquiritin apioside: m/*z* 135.0106, 119.0517; isosalipurposide: m/*z* 151.0048, 119.0511, Figure 4).

**Figure 5:**
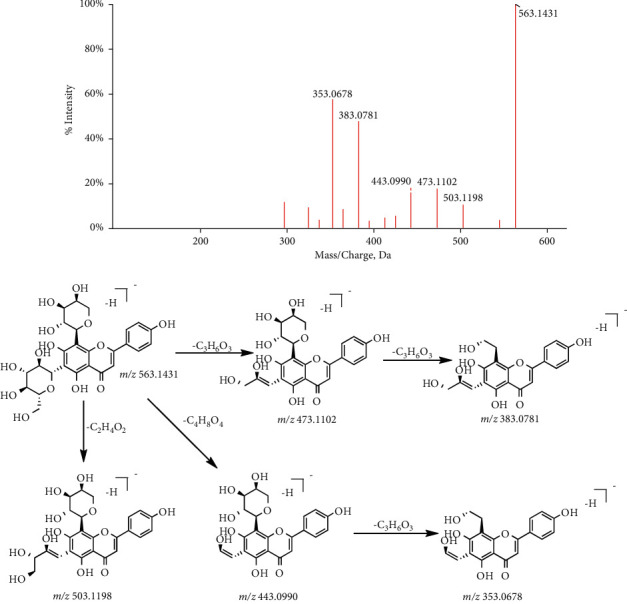
The fragmentation pathway of schaftoside.

*(3) C-Glycosyl Flavonoids.* In C-glycosides flavonoids, the glycosyl groups are mostly linked at the position of C_6_ or C_8_, or at both C_6_ and C_8_. Because the C–C bond of flavonoid C-glycosides is unstable, the ring-opening reaction of glycosyl groups mainly occurs in the process of cleavage. The continuous neutral loss of H_2_O(m/*z* 18.0106 Da) is dominant in the positive ion mode, and the glycosyl neutral loss is mainly in the negative ion mode, such as hexose neutral loss (C_4_H_8_O_4_ m/*z* 120.0423 or C_3_H_6_O_3_ m/*z* 90.0317) and pentose neutral loss (C_3_H_6_O_3_ m/*z* 90.0317 or C_2_H_4_O_2_ m/*z* 60.0211] [17]. Schaftoside(C_26_H_28_O_14_, [M-H]^−^, P30) is used as a reference to explain its cracking law. According to the information provided by the mass spectrogram, the parent ion of schaftoside is m/*z* 563.1431 and a series of characteristic ions are m/*z* 503.1198, 473.1102, 443.0990, 383.0781, and 353.0678, whose mass difference of each fragment ion is an integral multiple of CH_2_O (m/*z* 30.0106). The specific cracking process is shown in Figure 5. On the basis of the above rules, 17 flavonoid glycosides were identified, including 13 O-glycosyl flavonoids(labeled as P34, 35, 37, 38, 40, 43, 45, 55, 59, 66, 68, 70, 71) and 4 flavonoid C-glycosyl flavonoids(labeled as P24, 30, 44, 53).

#### 3.1.2. Identification of Triterpenoids

The basic skeleton of triterpenoids is connected by 6 isoprene structural units. Considering the skeleton with multiple six-membered rings in the structure is extremely stable and not easy to break bonds, higher collision energy is needed in the process of mass spectrometry pyrolysis. Triterpenes and their glycosides are easy to remove sugar groups (such as glucose) and ring-linked substituents, including OH (lost in the form of H_2_O, m/*z* 18.0106 Da), COOH(lost in the form of CO_2_, m/*z* 43.9898 Da), and other substituent groups [18].

**Figure 6:**
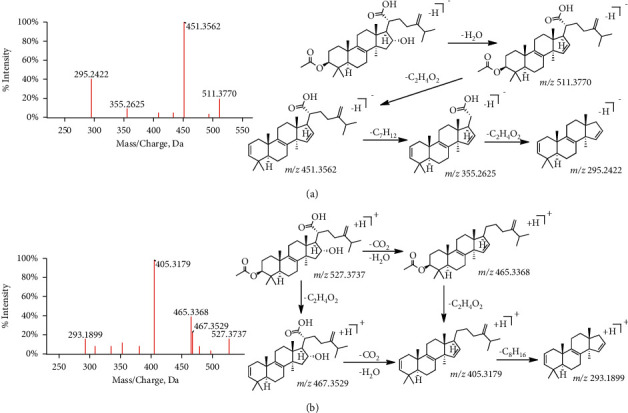
The fragmentation pathway of pachymic acid. The fragmentation pathway of (a) positive ion mode and (b) negative ion mode.

For our study, most of triterpenoids in the prescription mainly come from FL and GC. With reference to tumulosic acid (C_31_H_50_O_4_, [M-H]^−^, P130), the difference between parent ion and daughter ion is m/*z* 62.0004. That is to say, the compound loses CO_2_ and H_2_O at one time (parent ion-daughter ion: m/*z* 485.3639–423.3196). The following is to compare the differences of fragmentation mechanism between the positive and negative ion mode with pachymic acid (C_33_H_52_O_5_, P146). In the positive ion mode, when the collision energy is set to 40, the parent ion peak is not displayed, and the daughter ion is m/*z* 511.3770, 451.3562, 355.2625, and 295.2422. Among them, m/*z* 511.3770 is formed by removing H_2_O on the basis of the parent ion, and the subsequent daughter ions lose CH_3_COOH, branched-chain C_7_H_12_, and C_2_H_4_O_2_. However, in negative ion mode, when the collision energy is set to 40, basically only 527 single peak can be displayed (there is one peak at 465, and the intensity is only 2.37%). Increasing the collision energy to 80, the parent ion is at m/*z* 527.3737 and the daughter ion peaks are at m/*z* 467.3529, 465.3368, 405.3179, and 293.1899 in sequence. On the basis of the parent ion, on the one hand, it can lose CO_2_ and H_2_O to form the m/*z* 465.3368 ion. On the other hand, it can also lose the CH_3_COOH to form the m/*z* 467.3529 ion, which can continue to complete the loss of another group of ions, forming a 405.3179 peak. Finally, the branched-chain C_8_H_16_ was removed again to form the 293.1899 peak. The detailed cracking process is shown in Figure 6. According to the rules discussed above, a total of 30 compounds are inferred (labeled as P82, 86, 88, 89, 90, 91, 92, 102, 105, 106, 107, 108, 111, 121, 128, 129, 130, 133, 134, 136, 137, 138, 139, 142, 143, 144, 145, 146, 148, 149).

#### 3.1.3. Identification of Phenylpropanoids

**Figure 7:**
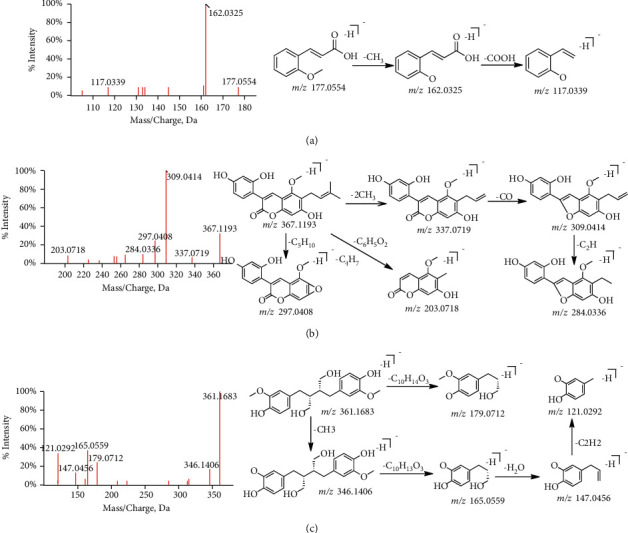
The fragmentation pathway of phenylpropanoids. The fragmentation pathway of (a) 2-methoxycinnamic acid, (b) glycycoumarin, and (c) secoisolariciresinol.

Phenylpropanoid compounds mainly include lignans, coumarins, phenylpropanoids, and their glycosides, which have different structural characteristics. Phenylpropanols are a kind of naturally occurring compound composed of benzene ring and three straight-chain carbon groups (C_6_–C_3_ groups). It generally has a phenol structure and is also a phenolic substance. Because caffeoyl groups often exist in its structure, the characteristic fragment ions of [M-H-C_9_H_6_O_3_] can often be obtained in the fragmentation of mass spectrometry. In addition, it is easy to lose CH_3_, H_2_O, CO, CO_2_, or COOH to obtain fragment ions with mass loss of 15, 18, 28, 44, and 45 Da [19, 20]. For example, compound 2-methoxycinnamic acid(C_10_H_10_O_3_, [M-H]^−^, P46) produced an m/*z* peak at 162.0326 upon the elimination of CH_3_, and this was followed by the loss of COOH groups in succession to give peaks at m/*z* 117.0339(Figure 7(a)).

The basic nuclear structure of coumarin is benzopyranone (cis-2-hydroxycinnamic acid lactone). According to the different substituents and connection modes, coumarin can be divided into simple coumarin, furocoumarins, pyranocoumarins, and other coumarins. Coumarins generally have many CO, CO_2_, OH, H_2_O, CH_3,_ and OCH_3_ connected to aromatic rings, so that a series of neutral ion peaks with continuous loss often appear. In addition, coumarins often have common functional groups such as isopentenyl, acetoxy, and 5-carbon unsaturated acyloxy groups, which are also the main characteristics of coumarin compounds [21, 22]. Glycycoumarin displayed the ions at m/*z* 367.1193 (C_21_H_19_O_6_, [M-H]^−^, P104), with the following diagnostic fragment ions provided by the MS/MS spectrum at m/*z* 337.0719 (C_19_H_13_O_6_, [M-H-2CH_3_]^−^), 309.0414 (C_18_H_13_O_5_, [M-H-2CH_3_–CO]^−^), 297.0408 (C_16_H_9_O_6_, [M-H-2CH_3_–C_3_H_4_]^−^), 284.0336 (C_16_H_12_O_5_, [M-H-2CH_3_–O–C_2_H]^−^), and 203.0718 (C_11_H_7_O_4_, [M-H–C_6_H_5_O_2_–C_4_H_7_]^−^) (Figure 7(b)).

Lignans are natural compounds of derivatives formed by the oxidative polymerization of phenylpropanoids, usually in the form of dimers, as well as a few number of trimers and tetramers. Most of them exist in free form in plant wood and resin, and also combine with glycosyl groups to form glycosides. As the phenylpropanoids polymers, the typical characteristic ion fragments of lignans are M/2 or M/3 peak, whose response intensity is relatively high. The other cleavage rules are consistent with phenylpropanols [23]. In the mass spectrogram of secoisolariciresinol (C_20_H_26_O_6_, [M-H]^−^, P64), it is observed that the parent ion is m/*z* 361.1683, with the daughter ion M/2–1 peak m/*z* 179.0712. The rest of the fragment ions, such as m/*z* 165.0559, 147.0456, and 121.0292, are formed by losing CH_3_, H_2_O, C_2_H_2_ in the case of monomers(Figure 7©). According to the fragmentation mechanism demonstrated, a total of 22 compounds are inferred (labeled as P13, 16, 17, 29, 42, 46, 48, 52, 54, 62, 63, 64, 75, 77, 80, 94, 98, 104, 113, 116, 117, 122).

#### 3.1.4. Identification of Organic Acids

**Figure 8:**
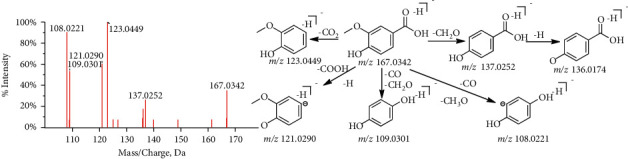
The fragmentation pathway of vanillic acid.

Organic acids are one kind of acidic compounds, which contain acidic functional groups, such as COOH, OH, SO_3_H, RSO_2_H, and RCOSH. Because there is less S element in TCM, they are mainly carboxylic acids and phenolic compounds. The response of these compounds in negative ion mode is better than that in positive, and it is easy to lose neutral molecules, such as CH_3_.H_2_O, CH_3_O, CO_2_, and COOH. When the compound contains ferulic acid or caffeic acid, the loss of feruloyl or caffeoyl C_9_H_6_O_3_ (146 Da) is also easy to occur [24, 25]. The prominent ion of vanillic acid (C_8_H_7_O_4_, m/*z* 167.0342, [M-H]^−^, P9) produced a series of characteristic ion at m/*z* 136.0174, 123.0449, 108.0221, which is caused by the loss of CH_3_O, CO_2_, and C_2_H_3_O_2_ (CH_3_O + CO) on the basis of molecular ion peaks (Figure 8). According to the rules discussed above, a total of 13 compounds are inferred (labeled as P2, 3, 9, 10, 11, 12, 14, 15, 19, 25, 26, 36, 79).

#### 3.1.5. Identification of the Other Types

In addition, by comparing the existing literature at home and abroad, exact molecular weight, mass spectrometry characteristics, and other related data, the study also preliminarily identified or inferred 21 species, including 5 alkaloids (labeled as P5, 6, 18, 22, 27), 6 lactones(labeled as P7, 8, 23, 61, 96, 127), 4 anthraquinones (labeled as P33, 56, 57, 78), 2 polysaccharides(labeled as P1, 4), and 4 other components(labeled as P31, 32, 95, 147) [26–30]. Given the large differences in the amounts of the various components, they are hereby grouped into other categories.

### 3.2. Biomarkers Screening and Validation by Network Pharmacology and Components Absorbed into Blood

**Figure 9:**
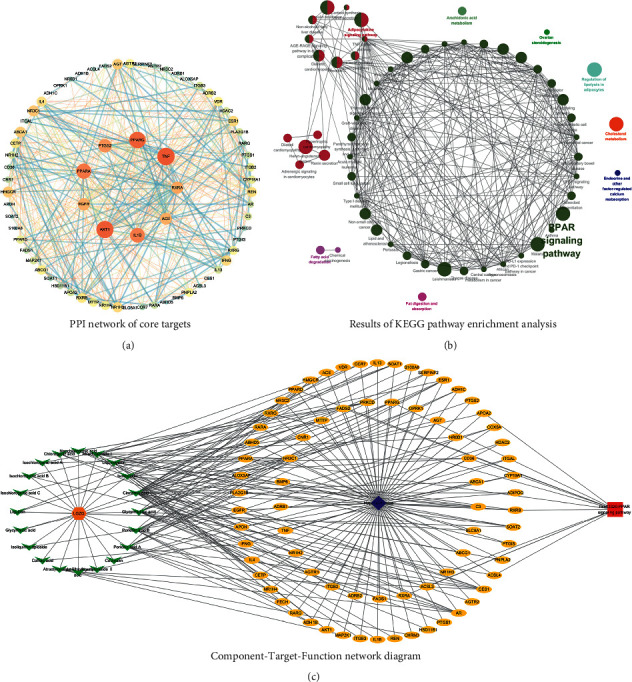
Network pharmacology prediction analysis. (a) PPI network of core target. (b) Result of KEGG pathway enrichment analysis. (c) Component target function network diagram.

**Figure 10:**
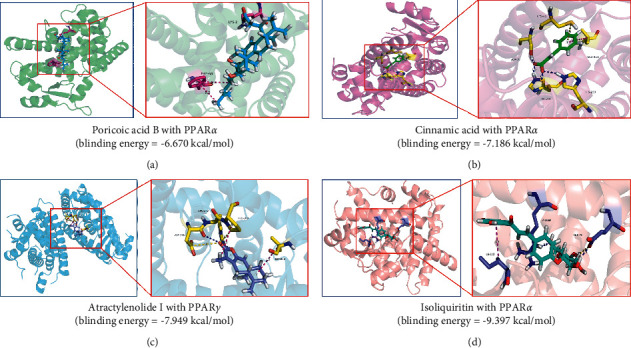
Result of molecular docking between typical chemical composition. (a) Poricoic acid B with PPAR*α* (binding energy = −6.670 kcal/mol). (b) Cinnamic acid with PPAR*α* (binding energy = −7.186 kcal/mol). (c) Atractylenolide I with PPAR*γ* (binding energy = −7.949 kcal/mol). (d) Isoliquiritin with PPAR*α* (binding energy = −9.397 kcal/mol).

A total of 284 potential targets related to the 149 identified compounds were obtained from BATMAN-TCM (http://bionet.ncpsb.org.cn/batman-tcm/) databases(with the score cutoff > 20). Through the keyword “Hyperlipidemias,” a total of 1,691 disease targets were obtained from DrugBank (https://go.drugbank.com/), OMIM (https://omim.org/), GeneCards (https://www. genecards.org/), and DisGeNET (https://www.disgenet.org/) databases. Through protein-protein interaction analysis, 71 targets with higher association were obtained as shown in Figure 9(a). The relevant parameters of the first 20 targets with a higher degree of integration are shown in Table 2. These reserved proteins were further imported into the ClueGo plugin of Cytoscape 3.7.2 software for KEGG enrichment analysis, and relatively important disease pathways were found as shown in Figure 9(b). Finally, the “component-target-function” network was visualized in Figure 9(c). There are a total of 20 compounds (including poricoic acid A, poricoic acid B, glycyrrhizic acid, glycyrrhetinic acid, liquiritin, isoliquiritin, liquiritigenin, isoliquiritin apioside, cinnamic acid, caffeic acid, neochlorogenic acid, chlorogenic acid, cryptochlorogenic acid, isochlorogenic acid A, isochlorogenic acid B, isochlorogenic acid C, atractylenolide I, atractylenolide II, atractylenolide III, and coumarin), 71 proteins, and 20 pathways involved. Of the 20 pathways, the score of PPAR pathway was the highest, which plays an important role in lipid metabolism. On this basis, we verify it by the method of molecular docking. The results show that the free energies of docking binding of 20 compounds are all greater than -6.471 kcal/mol. The typical components of the four TCM we selected are displayed in Figure 10. Besides, all the 20 compounds were detected in the rat serum by using UPLC-QQQ-MS/MS, which further verified that these compounds were proper biomarkers.

### 3.3. Quantification of the Major Constituents in LGZG by UPLC-QQQ-MS/MS

**Figure 11:**
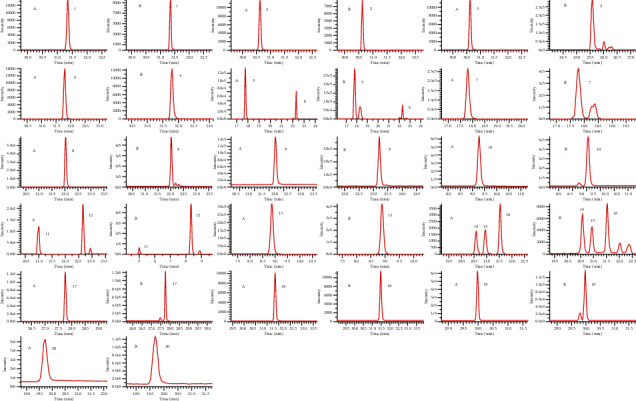
MRM chromatograms of reference substances (A) and samples (B). (1) Poricoic acid (A); (2) poricoic acid (B); (3) glycyrrhizic acid; (4) glycyrrhetinic acid; (5) liquiritin; (6) isoliquiritin; (7) liquiritigenin; (8) isoliquiritin apioside; (9) cinnamic acid; (10) caffeic acid; (11) neochlorogenic acid; (12) chlorogenic acid; (13) cryptochlorogenic acid; (14) isochlorogenic acid; (15) isochlorogenic acid (A); (16) isochlorogenic acid (C); (17) atractylenolide III; (18) atractylenolide (I); (19) atractylenolide II; (20) coumarin.

On the basis of the qualitative study and network pharmacology, the characteristic parameters of 20 main chemical components were investigated by the MRM analysis method, including parent ion, daughter ion, declustering potential, and collision energy. In order to satisfy the simultaneous quantification of multiple components, taking into account the response of different chemical components in MRM mode and the content differences in LGZG, the DP and CE of some chemical components were dynamically adjusted, as detailed in Table 3 [31–37]. Multiple reaction monitoring chromatograms of the control and sample solutions are shown in Figure 11.

#### 3.3.1. Method Validation

*(1) Calibration Curve, LOD, and LOQ*. According to the concentration (X) and peak area (Y) of each reference substance, the calibration curves were drawn and linear regressions were carried out, which showed good linearities(R^2^ > 0.9937). In addition, the LOD and LOQ values were determined, and the obtained results are presented in Table 4.

*(2) Precision, Repeatability, Stability, and Recovery*. The precision, repeatability, stability, and recovery of the quantitative method were investigated. The RSD of the precision of the reference substance and the sample did not exceed 4.29% and 5.02%, respectively. The repetitive result is less than 6.47%. The stability test results show that the LGZG sample solution is stable within 48 hours, and the RSD is less than 6.01%. The average recovery is between 96.22% and 104.19%. The results show that the established quantitative method is accurate, reliable, and reproducible and can be used to evaluate the quality of LGZG (Table 5).

#### 3.3.2. Sample Analysis

**Figure 12:**
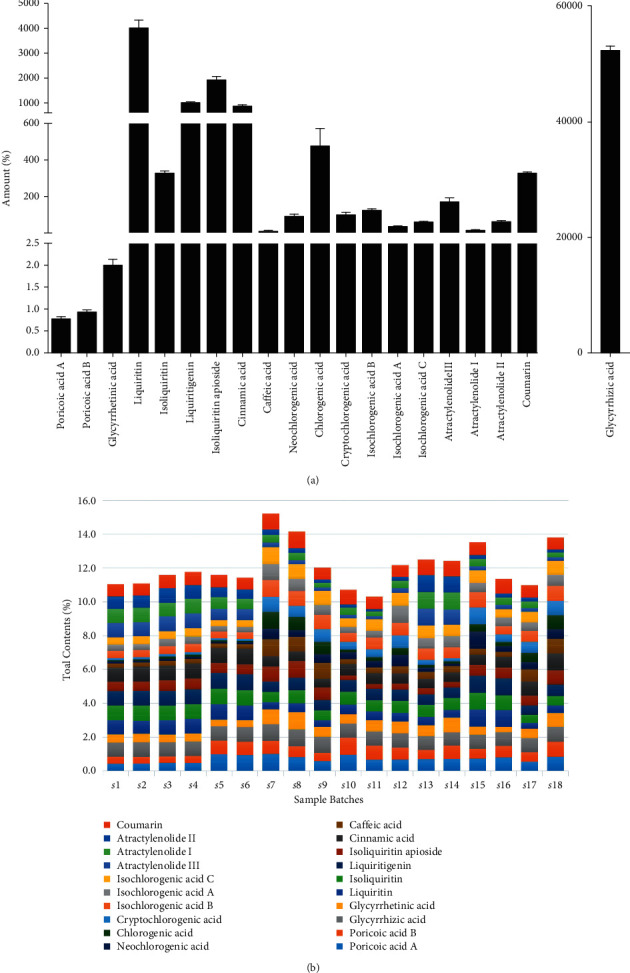
The quantitative study analysis results of LGZC. (a) Quantification results of 20 chemical components. (b) Total contents differences of 20 chemical components among the 18 batches.

The established quantitative method was used for the determination of 20 compounds in 18 batches of LGZG from different producing areas. The content ranges were poricoic acid A 0.46∼1.09 *μ*g/g, poricoic acid B 0.53∼1.43 *μ*g/g, glycyrrhizic acid 47.06∼60.36 mg/g, glycyrrhetinic acid 1.07∼3.32 *μ*g/g, liquiritin 2.03∼6.13 mg/g, isoliquiritin 204.17∼428.91 *μ*g/g, liquiritigenin 0.72 ∼1.30 mg/g, isoliquiritin apioside 0.82∼3.25 mg/g, cinnamic acid 0.58∼1.17 *μ*g/g, caffeic acid 6.07∼24.93 *μ*g/g, neochlorogenic acid 24.33∼252.05 *μ*g/g, chlorogenic acid 109.42–1386.51 *μ*g/g, cryptochlorogenic acid 25.65∼217.65 *μ*g/g, isochlorogenic acid A 20.98∼68.03 *μ*g/g, isochlorogenic acid B 75.52∼193.39 *μ*g/g, isochlorogenic acid C 34.37∼92.05 *μ*g/g, atractylenolide I 6.77∼25.56 *μ*g/g, atractylenolide II 16.95∼124.62 *μ*g/g, atractylenolide III 58.34∼339.74 *μ*g/g, and coumarin 281.17∼410.14 *μ*g/g (Figure 12, Table 6). The established determination method has been confirmed to be simple in operation, which also has the advantages of good precision, stability, repeatability, and recovery. It can provide a reference for the quality evaluation and quality control of the LGZG.

## 4. Conclusion

Based on UPLC-MS/MS and network pharmacology, this article established a research method of chemical composition research-network pharmacology predictive analysis-quality evaluation system. First of all, the 149 components of LGZG were preliminarily identified or inferred by UPLC-Q/TOF-MS, including flavonoids, triterpenoids, phenylpropanoids, organic acids, quinones, and other types of components. Then, a total of 20 biomarkers were screened by network pharmacological screening based on the presumed chemical composition. Finally, the quantitative determination method of biomarkers in LGZG was established by UPLC-QQQ-MS/MS, which covered all 4 herbal medicines in LGZG. Combined with statistical analysis, the quality evaluation system of LGZG was established. Following the results of this study, its pharmacological effects point to lipid metabolism-related targets and pathways. Therefore, the experimental group intends to carry out further studies on LGZG metabolomics and lipidomics to provide further data support for its pharmacodynamics and pharmacological mechanism.

## Figures and Tables

**Table 1:** Origin of 18 batch samples of TCM.

Batches	FL	GZ	BZ	GZ
**1**	YF18081902	YF18083004	YF18070702	YF18063004
**2**	YF18081902	YF18083004	YF18070702	YF18063004
**3**	YF18081902	YF18083004	YF18070702	YF18063004
**4**	YF18081902	YF18083004	YF18070702	YF18063004
**5**	YF18081902	YF18083004	YF18070702	YF18063004
**6**	YF18081902	YF18083004	YF18070702	YF18063004
**7**	YF18081914	GZ180321	YF18070603	YF18062905
**8**	YF18081914	GZ180321	YF18070603	YF18062905
**9**	YF18081914	GZ180321	YF18070603	YF18062905
**10**	FL180315	1710203	YF18070802	YF18062907
**11**	FL180315	1710203	YF18070802	YF18062907
**12**	FL180315	1710203	YF18070802	YF18062907
**13**	YF18081902	GZ180321	YF18070702	YF18062907
**14**	YF18081902	GZ180321	YF18070702	YF18062907
**15**	YF18081914	1710203	YF18070802	YF18063004
**16**	YF18081914	1710203	YF18070802	YF18063004
**17**	FL180315	YF18083004	YF18070603	YF18062905
**18**	FL180315	YF18083004	YF18070603	YF18062905

**Table 2:** The first 20 key targets of LGZG decoction in treating hyperlipidemias.

No.	Target name	Betweenness centrality	Closeness centrality	Degree
1	TNF	0.1205	0.7204	41
2	AKT1	0.1198	0.7053	40
3	PPARG	0.1057	0.6907	38
4	PPARA	0.0951	0.6569	33
5	IL1B	0.0514	0.6505	32
6	PTGS2	0.0414	0.6091	28
7	RXRA	0.0320	0.6036	24
8	EGFR	0.0230	0.5877	24
9	ACE	0.0371	0.5982	23
10	HMGCR	0.0370	0.5776	20
11	NR3C1	0.0147	0.5492	20
12	ABCA1	0.0240	0.5678	19
13	AGT	0.0320	0.5678	19
14	NR1H3	0.0090	0.5630	18
15	IFNG	0.0144	0.5678	18
16	ESR1	0.0104	0.5194	18
17	REN	0.0139	0.5537	17
18	IL4	0.0105	0.5583	17
19	CETP	0.0142	0.5194	16
20	RXRB	0.0072	0.5276	16

**Table 3:** Mass spectrometry data of 20 chemical constituents.

Chemical components	*t*_*R*_ (min)	Detection mode	Parent ion (m/*z*)	Daughter ion (m/*z*)	Declustering potential (V)	Collision energy (V)
Poricoic acid A	31.36	M-H	497.2	423.3	−44.00	−214.00
Poricoic acid B	30.64	M-H	483.4	409.4	−44.40	−203.00
Glycyrrhizic acid	25.53	M-H	821.4	350.8	−78.00	−276.00
Glycyrrhetinic acid	31.80	M-H	469.2	425.4	−49.90	−247.00
Liquiritin And isoliquiritin	17.81 22.31	M-H	417.2	255.0	−47.00	−215.00
Liquiritigenin	17.81	M-H	255.0	134.7	−43.00	−178.00
Isoliquiritin apioside	22.04	M-H	549.2	254.8	−53.00	−284.00
Cinnamic acid	23.23	M-H	146.6	102.8	−14.08	−148.86
Caffeic acid	9.31	M-H	178.6	134.6	−23.88	−104.01
Neochlorogenic acid and chlorogenic acid	5.02 8.35	M-H	353.0	190.6	−22.91	−152.04
Cryptochlorogenic acid	8.91	M-H	353.1	172.6	−22.43	−114.04
Isochlorogenic acid A, B, and C	20.96 20.59 21.54	M-H	515.1	353.1	−37.00	−180.00
Atractylenolide III	27.76	M-H	247.0	202.7	−22.95	−147.87
Atractylenolide I	31.56	M + H	231.1	129.0	37.83	136.00
Atractylenolide II	29.98	M + H	233.3	215.2	20.00	133.00
Coumarin	19.70	M + H	147.2	91.0	28.03	109.00

**Table 4:** Regression equations, correlation coefficients, linear ranges, LODs, and LOQs of 20 chemical constituents.

<!-Col Count:6-->Chemical components	Calibration curve	*R* ^2^	Linearity range (*μ*g/mL)	LOD (ng/mL)	LOQ (ng/mL)
Poricoic acid A	*Y* = 72391*X* − 5034.7	0.9994	0.002∼0.21	0.199	0.665
Poricoic acid B	*Y* = 68768*X* − 7167.6	0.9989	0.002∼0.22	0.177	0.591
Glycyrrhizic acid	*Y* = 229221*X* − 89186	0.9970	2.829∼282.9	499.2	1664
Glycyrrhetinic acid	*Y* = 76305*X* + 1723.8	0.9995	0.003∼0.32	0.461	1.538
Liquiritin	*Y* = 1E + 06X − 97606	0.9952	3.264∼326.4	90.08	300.3
Isoliquiritin	*Y* = 472548*X* − 40833	0.9988	0.042∼42.12	12.74	42.46
Liquiritigenin	*Y* = 1E + 06X + 1E + 06	0.9959	2.465∼61.63	28.94	96.48
Isoliquiritin apioside	*Y* = 601173*X* − 225511	0.9950	0.536∼53.62	26.81	89.37
Cinnamic acid	*Y* = 751463*X* + 182882	0.9939	0.768∼76.80	157.8	526.0
Caffeic acid	*Y* = 5E + 06X + 1E + 06	0.9945	0.017∼1.697	1.858	6.192
Neochlorogenic acid	*Y* = 3E + 06X − 953535	0.9963	0.166∼16.55	5.623	18.72
Chlorogenic acid	*Y* = 4E + 06X − 961985	0.9978	0.162∼16.15	2.525	8.416
Cryptochlorogenic acid	*Y* = 3E + 06X + 88218	0.9960	0.055∼5.528	1.229	4.096
Isochlorogenic acid A	*Y* = 31732*X* − 10255	0.9971	0.039∼3.894	4.314	14.38
Isochlorogenic acid B	*Y* = 33.14X + 17963	0.9997	0.024∼2.430	8.404	28.01
Isochlorogenic acid C	*Y* = 48826*X* − 11262	0.9937	0.034∼3.353	7.288	24.29
Atractylenolide III	*Y* = 628413*X* − 27614	0.9996	0.060∼6.000	3.379	11.26
Atractylenolide I	*Y* = 78592*X* − 1874.8	0.9995	0.033∼3.300	18.80	62.67
Atractylenolide II	*Y* = 345026*X* + 43919	0.9986	0.094∼9.400	13.95	46.51
Coumarin	*Y* = 731434*X* − 14528	0.9998	0.274∼27.36	241.41	804.7

**Table 5:** The results of precision, reproducibility, stability, and recovery of 20 chemical constituents.

Chemical components	Precision (RSD/%)		Reproducibility (RSD/%)	Stability (RSD/%)	Recovery	
	Reference	Sample			Average (%)	RSD (%)
Poricoic acid A	1.77	4.22	0.81	4.42	99.25	2.42
Poricoic acid B	2.70	2.96	2.83	5.41	102.24	1.06
Glycyrrhizic acid	1.25	1.36	3.28	4.79	103.39	3.23
Glycyrrhetinic acid	2.85	2.95	3.70	4.25	98.24	1.46
Liquiritin	2.44	2.27	5.54	5.16	100.30	2.60
Isoliquiritin	3.55	2.44	2.16	4.29	99.75	1.84
Liquiritigenin	1.80	0.57	2.45	1.52	96.22	3.11
Isoliquiritin apioside	3.67	1.83	5.51	1.98	100.95	1.69
Cinnamic acid	2.94	3.38	2.79	5.10	99.66	2.21
Caffeic acid	2.17	1.07	3.42	2.60	102.35	1.50
Neochlorogenic acid	2.83	1.69	1.53	5.24	98.99	1.83
Chlorogenic acid	3.40	3.46	2.40	5.78	96.98	2.41
Cryptochlorogenic acid	2.27	1.54	3.94	4.98	99.61	1.99
Isochlorogenic acid A	1.60	4.42	2.99	2.72	104.19	1.88
Isochlorogenic acid B	4.29	3.75	2.44	3.73	96.84	2.99
Isochlorogenic acid C	4.07	1.79	4.70	5.55	102.26	1.34
Atractylenolide III	2.78	3.54	3.34	3.86	101.39	1.45
Atractylenolide I	3.23	5.02	6.15	6.01	99.90	2.81
Atractylenolide II	1.95	2.08	5.79	6.00	97.82	1.22
Coumarin	3.73	2.22	6.47	5.98	98.13	1.16

**Table 6:** Determination of 20 chemical constituents of 18 batches of LGZG samples (*μ*g/g).

Chemical components	Sample batches																	
	1	2	3	4	5	6	7	8	9	10	11	12	13	14	15	16	17	18
Poricoic acid A	0.46	0.46	0.52	0.52	1.07	1.02	1.09	0.89	0.63	1.05	0.72	0.74	0.75	0.78	0.80	0.88	0.59	0.91
Poricoic acid B	0.60	0.57	0.53	0.57	1.16	1.13	1.11	0.92	0.69	1.43	1.18	0.98	0.78	1.12	0.82	0.96	0.80	1.26
Glycyrrhizic acid	50231	51954	50381	51863	51073	52301	59407	60362	58091	50475	51238	52194	49175	47062	49828	49119	50978	53611
Glycyrrhetinic acid	1.60	1.69	1.54	1.55	1.29	1.35	2.91	3.32	1.96	1.83	2.22	2.29	2.17	2.85	1.64	1.07	1.86	2.66
Liquiritin	5169	4763	5202	5344	5595	5313	2549	3275	2439	3457	3153	3119	2946	2838	6128	6009	2025	2805
Isoliquiritin	366.8	377.2	364.6	367.2	397.7	387.2	263.6	334.3	239.4	326.3	284.8	304.0	301.9	305.7	428.9	376.9	204.2	240.5
Liquiritigenin	1123	1143	1142	1159	1193	1228	795.6	947.9	816.0	920.4	871.4	872.0	816.1	807.5	1302	1298	722.8	876.7
Isoliquiritin apioside	1792	1811	2029	2068	1941	1912	2914	3249	2427	822.8	958.3	1179	1198	1076	2078	2076	1869	2735
Cinnamic acid	972.2	1031	1021	1048	1051	1096	712.2	669.7	586.7	797.8	740.6	687.6	640.9	648.8	713.7	753.2	983.8	1168
Caffeic acid	6.32	6.07	7.29	6.93	6.54	6.72	24.93	21.21	23.73	7.29	8.15	10.46	10.53	9.60	8.92	7.26	18.14	21.78
Neochlorogenic acid	26.82	26.40	31.82	32.31	24.33	25.57	156.0	104.4	129.8	102.5	103.6	176.3	59.84	38.47	252.1	87.21	102.2	144.0
Chlorogenic acid	117.8	109.4	162.9	159.3	108.7	106.0	1386	1057	996.8	232.7	279.4	544.2	251.5	166.3	604.7	321.3	780.8	1139
Cryptochlorogenic acid	27.96	27.65	33.83	32.99	25.80	25.59	194.4	144.1	165.3	97.69	104.4	163.9	62.60	42.32	217.7	97.68	141.8	187.2
Isochlorogenic acid B	82.45	82.62	90.29	94.60	78.78	75.52	193.4	169.2	164.1	100.3	133.1	143.2	130.2	124.7	175.2	93.05	126.0	170.0
Isochlorogenic acid A	25.10	22.72	28.28	28.63	20.98	21.51	63.67	49.13	38.87	23.79	27.10	68.03	41.13	44.32	37.00	35.57	33.94	44.34
Isochlorogenic acid C	39.99	44.84	42.58	46.39	34.37	37.20	92.05	78.38	77.72	48.08	63.54	71.27	70.17	63.03	68.58	45.84	58.38	76.24
Atractylenolide III	287.6	281.9	299.8	298.7	226.9	226.4	94.56	83.27	71.46	69.80	58.34	94.49	339.7	307.5	82.35	86.96	75.49	72.11
Atractylenolide I	21.22	21.68	20.23	21.88	17.63	15.50	11.20	11.00	6.77	10.88	7.40	11.34	25.11	25.56	10.80	11.26	9.66	7.07
Atractylenolide II	92.37	91.16	109.5	101.6	72.56	68.78	41.33	34.77	24.90	23.45	16.95	28.20	124.6	119.2	28.51	27.71	30.52	23.52
Coumarin	302.9	294.4	323.0	320.8	300.0	283.1	390.0	410.1	286.5	354.6	302.7	281.2	374.8	373.3	312.3	361.3	297.3	289.7

## Data Availability

The figure and table data used to support the findings of this study are included within the article.

## Supplementary Materials

Supplementary Materials Table S1: characterization of chemical components derived from LGZG by UPLC-Q/TOF-MS/MS.
