# Supplementary material for: Phytochemical Analysis Using UPLC-MS/MS Combined with Network Pharmacology Methods to Explore the Biomarkers for the Quality Control of Lingguizhugan Decoction
Source: Evid Based Complement Alternat Med. 2021 Dec 22;2021:7849032. doi: 10.1155/2021/7849032 (PMC8716202; doi:10.1155/2021/7849032)
Supplement: Supplementary Materials — Table S1: characterization of chemical components derived from LGZG by UPLC-Q/TOF-MS/MS. [file 7849032.f1.doc]

**Tab.S1 Characterization of chemical components derived from LGZG by UPLC-Q/TOF-MS/MS**

| **NO.** | **Ion Type** | **RT**  **(min)** | **Formula** | **Extraction Mass**  **(m/z)** | **Experimental Mass**  **(m/z)** | **Error**  **(ppm)** | | | | | | **Fragement Ion(m/z)** | | | | | | | | | | | | | | | | | | | **Identification** | | | | | | | | | | | | | | **Type** | | | | |
| --- | --- | --- | --- | --- | --- | --- | --- | --- | --- | --- | --- | --- | --- | --- | --- | --- | --- | --- | --- | --- | --- | --- | --- | --- | --- | --- | --- | --- | --- | --- | --- | --- | --- | --- | --- | --- | --- | --- | --- | --- | --- | --- | --- | --- | --- | --- | --- | --- | --- |
| 1 | [M-H]^-^ | 0.83 | C_12_H_22_O_11_ | 341.1090 | 341.1090 | 0.2 | | | | | | 341.1108(M-H,C_12_H_21_O_11_);179.0565(M-H-C_6_H_10_O_5_,C_6_H_11_O_6_);161.0461 (M-H-C_6_H_12_O_6_,C_6_H_9_O_5_);119.0354(M-H-C_6_H_10_O_5_-C_2_H_4_O_2_,C_4_H_7_O_4_); | | | | | | | | | | | | | | | | | | | Lactose | | | | | | | | | | | | | | O | | | | |
| 2 | [M-H]^-^ | 0.84 | C_7_H_12_O_6_ | 191.0561 | 191.0562 | 0.4 | | | | | | 191.0569(M-H,C_7_H_11_O_6_);111.0097(M-H-CO_2_-2H_2_O,C_6_H_7_O_2_) | | | | | | | | | | | | | | | | | | | Quinic acid | | | | | | | | | | | | | | OA | | | | |
| 3 | [M-H]^-^ | 0.89 | C_4_H_6_O_5_ | 133.0143 | 133.0143 | 0.7 | | | | | | 133.0149(M-H,C_4_H_5_O_5_);115.0039(M-H-H_2_O,C_4_H_3_O_4_) | | | | | | | | | | | | | | | | | | | L-Malic acid | | | | | | | | | | | | | | OA | | | | |
| 4 | [M-H]^-^ | 1.09 | C_24_H_42_O_21_ | 665.2146 | 665.2144 | -0.3 | | | | | | 665.2164(M-H,C_24_H_41_O_21_);341.1112(M-H-2C_6_H_10_O_5_,C_12_H_21_O_11_);179.0570 (M-H-3C_6_H_10_O_5_,C_6_H_11_O_6_);161.0467(M-H-3C_6_H_10_O_5_-H_2_O,C_6_H_9_O_5_) | | | | | | | | | | | | | | | | | | | Nistose | | | | | | | | | | | | | | O | | | | |
| 5 | [M+H]^+^ | 1.10 | C_10_H_13_N_5_O_5_ | 284.0990 | 284.0990 | 0.1 | | | | | | 284.1232(M+H,C_10_H_14_N_5_O_5_)152.0566(M+H-C_5_H_8_O_4_,C_5_H_6_N_5_O);135.0298 (M+H-C_5_H_8_O_4_-NH_3_,C_5_H_3_N_4_O) | | | | | | | | | | | | | | | | | | | 2-Hydroxyadenosine | | | | | | | | | | | | | | AL | | | | |
|  | [M-H]^-^ | 1.15 | C_10_H_13_N_5_O_5_ | 282.0845 | 282.0845 | 0.3 | | | | | | 282.0847(M-H, C_10_H_12_N_5_O_5_); 150.0427(M-H-C_5_H_8_O_4_, C_5_H_4_N_5_O); 133.0161 (M-H-C_5_H_8_O_4_-NH_3_,C_5_HN_4_O);108.0204( M-H-C_5_H_8_O_4_-CH_2_N_2_, C_4_H_2_N_3_O); | | | | | | | | | | | | | | | | | | |  |  |  |  |  |  |  |  |  |  |  |  |  |  |  |  |  |  |  |
| 6 | [M+H]^+^ | 1.10 | C_10_H_13_N_5_O_4_ | 268.1040 | 268.1038 | -0.9 | | | | | | 268.1031(M+H, C_10_H_14_N_5_O_4_); 136.0614(M+H-C_5_H_8_O_4_, C_5_H_6_N_5_); 119.0345(M+H-C_5_H_8_O_4_-NH_3_,C_5_H_3_N_4_) | | | | | | | | | | | | | | | | | | | Adenosine | | | | | | | | | | | | | | AL | | | | |
| 7 | [M+H]^+^ | 1.41 | C_11_H_14_O_5_ | 227.0914 | 227.0916 | 0.8 | | | | | | 227.1386(M+H, C_11_H_15_O_5_); 167.0702(M+H-C_2_H_4_O_2_, C_9_H_11_O_3_); 149.0611 (M+H -C_2_H_4_O_2_-H_2_O, C_9_H_9_O_2_); 137.0593(M+H-C_2_H_4_O_2_-CH_2_O, C_8_H_9_O_2_); 131.0481 (M+H-C_2_H_4_O_2_-2H_2_O, C_9_H_7_O); 123.0432(M+H-C_2_H_4_O_2_-CO_2_, C_8_H_11_O); | | | | | | | | | | | | | | | | | | | Genipin | | | | | | | | | | | | | | L | | | | |
| 8 | [M-H]^-^ | 1.51 | C_10_H_12_O_4_ | 195.0663 | 195.0663 | 0 | | | | | | 165.0194(M-H-2CH_3_, C_8_H_5_O_4_); 137.0243(M-H-2CH_3_-CO, C_7_H_5_O_3_); | | | | | | | | | | | | | | | | | | | Cantharidin | | | | | | | | | | | | | | L | | | | |
| 9 | [M-H]^-^ | 2.19 | C_8_H_8_O_4_ | 167.0350 | 167.0351 | 0.5 | | | | | | 167.0342(M-H,C_8_H_7_O_4_);137.0252(M-H-CH_2_O, C_7_H_5_O_3_);136.0174(M-H -CH_3_O, C_7_H_4_O_3_);123.0449(M-H-CO_2_, C_7_H_7_O_2_); 121.0290(M-H-CH_2_O_2_, C_7_H_5_O_2_); 109.0301(M-H-CO-CH_2_O, C_6_H_5_O_2_);108.0221(M-H-CO-CH_3_O, C_6_H_4_O_2_) | | | | | | | | | | | | | | | | | | | Vanillic acid | | | | | | | | | | | | | | OA | | | | |
| 10 | [M-H]^-^ | 2.68 | C_7_H_6_O_4_ | 153.0193 | 153.0197 | 0.4 | | | | | | 153.0209(M-H,C_7_H_5_O_4_);109.0302(M-H-CO_2_,C_6_H_5_O_2_);  108.0221(M-H-COOH,C_6_H_4_O_2_) | | | | | | | | | | | | | | | | | | | 3,4-Dihydroxybenzoic acid | | | | | | | | | | | | | | OA | | | | |
| 11 | [M-H]^-^ | 2.99 | C_15_H_20_O_10_ | 359.0983 | 359.0981 | -0.3 | | | | | | 197.0415(M-H-C_6_H_10_O_5_, C_9_H_9_O_5_);182.0227(M-H-C_6_H_10_O_5_-CH_3_, C_8_H_6_O_5_);138.0332 (M-H-C_6_H_10_O_5_-CH_3_-CO_2_, C_7_H_6_O_3_);123.0091(M-H-C_6_H_10_O_5_-CH_3_-CO_2_-CH_3_, C_6_H_3_O_3_) | | | | | | | | | | | | | | | | | | | | | | | | | | Glucosyringic acid | | | | | | | OA | | | | |
| 12 | [M-H]^-^ | 3.28 | C_16_H_18_O_9_ | 353.0878 | 353.0877 | -0.2 | | | | | | 353.0859(M-H, C_16_H_17_O_9_);191.0570(M-H-C_9_H_6_O_3_, C_7_H_11_O_6_); 179.0360(M-H-C_7_H_10_O_5_,C_9_H_7_O_4_);135.0460(M-H-C_7_H_10_O_5_-CO_2_,C_8_H_7_O_2_) | | | | | | | | | | | | | | | | Neochlorogenic acid^*^ | | | | | | | | | | | | | | | | | P | | | | |
| 13 | [M-H]^-^ | 4.38 | C_7_H_6_O_3_ | 137.0244 | 137.0245 | 0.5 | | | | | | 137.0247(M-H,C_7_H_5_O_3_);136.0166(M-2H,C_7_H_4_O_3_);  119.0147(M-H-H_2_O,C_7_H_3_O_2_);108.0218(M-H-CHO,C_6_H_4_O_2_) | | | | | | | | | | | | | | | | | | | Protocatechuic aldehyde | | | | | | | | | | | | | | OA | | | | |
| 14 | [M-H]^-^ | 4.56 | C_9_H_10_O_3_ | 165.0557 | 165.0564 | 1 | | | | | | 165.0554(M-H,C_9_H_9_O_3_);147.8885(M-H-H_2_O ,C_9_H_7_O_2_);  121.0653(M-H-CO_2_,C_8_H_9_O);119.0498(M-H-CO_2_-H_2_,C_8_H_7_O) | | | | 3-(2-Hydroxyphenyl)propionic acid | | | | | | | | | | | | | | | | | | | | | | | | | | | | | OA | | | | |
| 15 | [M-H]^-^ | 7.02 | C_16_H_18_O_9_ | 353.0878 | 353.0876 | -0.7 | | | | | | 191.0568(M-H-C_9_H_6_O_3_,C_7_H_11_O_6_) | | | | Chlorogenic acid^*^ | | | | | | | | | | | | | | | | | | | | | | | | | | | | | | | P | | |
| 16 | [M-H]^-^ | 7.88 | C_9_H_8_O_4_ | 179.0350 | 179.0349 | -0.4 | | | | | | 179.0149(M-H, C_9_H_7_O_4_);135.0459(M-H-CO_2_, C_8_H_7_O_2_) | | | | | | | | | | | | | | | | | | | Caffeic acid^*^ | | | | | | | | | | | | | | OA | | | | |
| 17 | [M-H]^-^ | 8.50 | C_16_H_18_O_9_ | 353.0878 | 353.0877 | 0.2 | | | | | | 353.0915(M-H, C_16_H_17_O_9_);191.0571(M-H-C_9_H_6_O_3_,C_7_H_11_O_6_);179.0348 (M-H -C_7_H_10_O_5_,C_9_H_7_O_4_);173.0449(M-H-C_9_H_6_O_3_-H_2_O,C_7_H_9_O_5_);161.0249(M-H -C_7_H_10_O_5_-H_2_O,C_9_H_5_O_3_);135.0447(M-H-C_7_H_10_O_5_-CO_2_,C_8_H_7_O_2_) | | | | | | | | | | | | | | | | | | | Cryptochlorogenic acid^*^ | | | | | | | | | | | | | | P | | | | |
| 18 | [M+H]^+^ | 8.75 | C_18_H_19_NO_4_ | 314.1387 | 314.1388 | 0.4 | | | | | | 297.1135(M+H-NH_3_, C_18_H_17_O_4_) ;282.0880(M+H-NH_3_-CH_3_, C_17_H_14_O_4_) ; 267.0649 (M+H-NH_3_-CH_2_O, C_17_H_15_O_3_); 265.0876(M+H-NH_3_-CH_2_-H_2_O, C_17_H_13_O_3_); 222.0682(M+H-NH_3_-CH_2_-H_2_O-C_2_H_3_-O, C_15_H_10_O_2_) | | | | | | | | | | | | | | | | | | | Norisoboldine | | | | | | | | | | | | | | AL | | | | |
| 19 | [M-H]^-^ | 8.89 | C_9_H_10_O_5_ | 197.0456 | 197.0455 | -0.3 | | | | | | 197.0454(M-H, C_9_H_9_O_5_);182.0251(M-H-CH_3_, C_8_H_6_O_5_); 166.9980(M-H-C_2_H_6_,C_7_H_3_O_5_);123.0091(M-H-C_2_H_6_-CO_2_, C_6_H_3_O_3_) | | | | | | | | | | | | | | | | | | | Ethyl gallate | | | | | | | | | | | | | | OA | | | | |
| 20 | [M-H]^-^ | 9.30 | C_30_H_26_O_12_ | 577.1352 | 577.1351 | -0.1 | | | | | | 577.1364(M-H,C_30_H_25_O_12_);451.1035(M-H-C_6_H_4_O_2_-H_2_O,C_24_H_19_O_9_);425.0885(M-H-C_6_H_4_O_2_-H_2_O-C_2_H_2_,C_22_H_17_O_9_);407.0780(M-H-C_6_H_4_O_2_-H_2_O-C_2_H_2_-H_2_O,C_22_H_15_O_8_);289.0646(M-H-C_15_H_12_O_6_,C_15_H_13_O_6_);125.0248(M-H-C_15_H_12_O_6_-C_9_H_8_O_3_,C_6_H_5_O_3_) | | | | | | | | | | | | | | | | | | | Procyanidin B1 | | | | | | | | | | | | | | F | | | | |
| 21 | [M-H]^-^ | 10.00 | C_15_H_12_O_6_ | 287.0561 | 287.0560 | -0.3 | | | | | | 287.0574(M-H, C_15_H_11_O_6_);269.0439(M-H-H_2_O,C_15_H_9_O_5_);259.0639(M-H -CO,C_14_H_11_O_5_);243.0297(M-H-CO-O,C_14_H_11_O_4_);175.0408(M-H-CO-C_4_H_4_O_2_, C_10_H_7_O_3_);137.0244(M-H-C_8_H_6_O_3_,C_7_H_5_O_3_);125.0246(M-H-C_9_H_6_O_3_,C_6_H_5_O_3_); 121.0297(M-H-C_8_H_6_O_3_-O,C_7_H_5_O_2_);109.0298(M-H-C_8_H_6_O_3_-CO,C_6_H_5_O_2_) | | | | | | | | | | | | | | | | | | | Dihydrokaempferol | | | | | | | | | | | | | | F | | | | |
| 22 | [M+H]^+^ | 10.09 | C_17_H_20_N_4_O_6_ | 377.1456 | 377.1454 | -0.4 | | | | | | 377.1480(M+H,C_17_H_21_N_4_O_6_);359.1358(M+H-H_2_O,C_17_H_19_N_4_O_5_);243.0883  (M+H-C_5_H_10_O_4_,C_12_H_11_N_4_O_2_) ;200.0826(M+H-C_5_H_10_O_4_-CONH,C_11_H_10_N_3_O); 172.0889(M+H-C_5_H_10_O_4_-CONH-CO,C_10_H_10_N_3_) | | | | | | | | | | | | | | | | | | | Vitamin B2 | | | | | | | | | | | | | | AL | | | | |
| 23 | [M+H]^+^ | 10.17 | C_15_H_16_O_4_ | 261.1121 | 261.1122 | 0.1 | | | | | | 261.1306(M+H,C_15_H_17_O_4_);217.1222(M+H-CO_2_,C_14_H_17_O_2_);  199.1125(M+H-CO_2_-H_2_O,C_14_H_15_O);184.0869(M+H-CO_2_-H_2_O-CH_3_,C_13_H_12_O) | | | | | | | | | | | | | | | | | | | Linderane | | | | | | | | | | | | | | L | | | | |
| 24 | [M+H]^+^ | 10.45 | C_27_H_30_O_15_ | 595.1658 | 595.1661 | 0.6 | | | | | | 595.1651(M+H, C_27_H_31_O_15_);577.1564(M+H-H_2_O, C_27_H_29_O_14_); 559.1473(M+H-2H_2_O, C_27_H_27_O_13_); 541.1338(M+H-3H_2_O, C_27_H_25_O_12_); 481.1128(M+H-3H_2_O-C_2_H_4_O_2_, C_25_H_21_O_10_); 421.0922(M+H-3H_2_O-2C_2_H_4_O_2_, C_23_H_17_O_8_); 325.0715 (M+H -4C_2_H_4_O_2_-CH_2_O, C_18_H_13_O_6_);295.0603(M+H-5C_2_H_4_O_2_, C_17_H_11_O_5_) | | | | | | | | | | | | | | | | | | | Vicenin II | | | | | | | | | | | | | | F | | | | |
| 25 | [M-H]^-^ | 10.68 | C_9_H_8_O_3_ | 163.0401 | 163.0402 | 1 | | | | | | 163.0143(M-H, C_9_H_7_O_3_)119.0503(M-H-CO_2_, C_8_H_7_O);  117.0359(M-H-CH_2_O_2_, C_8_H_5_O) | | | | | | | | | | | | | | | | | | | 2-Hydroxycinnamic acid | | | | | | | | | | | | | | OA | | | | |
| 26 | [M-H]^-^ | 10.68 | C_9_H_8_O_3_ | 163.0401 | 163.0402 | 1 | | | | | | 163.0143(M-H, C_9_H_7_O_3_),119.0503(M-H-CO_2_,C_8_H_7_O) | | | | | | | | | | | Ttrans-4-Hydroxycinnamic acid | | | | | | | | | | | | | | | | | | | | | | OA | | | | |
| 27 | [M+H]^+^ | 10.93 | C_19_H_23_NO_4_ | 330.1700 | 330.1701 | 0.2 | | | | | | 330.1699(M+H, C_19_H_24_NO_4_) ;299.1281(M+H-CH_3_O, C_18_H_21_NO_3_);207.0786 (M+H-C_7_H_7_O_2_, C_12_H_17_NO_2_);192.1014(M+H-C_7_H_7_O_2_-CH_3_, C_11_H_14_NO_2_) | | | | | | | | | | | | | | | | | | | Sinomenine | | | | | | | | | | | | | | AL | | | | |
| 28 | [M-H]^-^ | 11.13 | C_45_H_38_O_18_ | 865.1985 | 865.1981 | -0.3 | | | | | | 865.2025(M-H, C_45_H_37_O_18_);577.1380(M-H-C_15_H_12_O_6_, C_30_H_25_O_12_);289.0725 (M-H-2C_15_H_12_O_6_, C_15_H_13_O_6_);125.0248(M-H-2C_15_H_12_O_6_-C_9_H_8_O_3_, C_6_H_5_O_3_) | | | | | | | | | | | | | | | | | | | Procyanidin C1 | | | | | | | | | | | | | | F | | | | |
| 29 | [M+H]^+^ | 11.54 | C_10_H_8_O_4_ | 193.0495 | 193.0494 | -0.5 | | | | | | 193.0497(M+H, C_10_H_9_O_4_); 178.0255(M+H-CH_3_,C_9_H_6_O_4_); 150.0310(M+H-CH_3_-CO,C_8_H_6_O_3_); 133.0280(M+H-CH_3_-CO-OH, C_8_H_5_O_2_); 122.0360(M+H-CH_3_-2CO,C_7_H_6_O_2_); 105.0326(M+H-CH_3_-2CO-OH, C_7_H_5_O) | | | | | | | | | | | | | | | | | | | Scopoletin | | | | | | | | | | | | | | P | | | | |
| 30 | [M+H]^+^ | 11.83 | C_26_H_28_O_14_ | 565.1552 | 565.1550 | 0 | | | | | | 565.1550(M+H, C_26_H_29_O_14_);547.1466(M+H-H_2_O,C_26_H_27_O_13_); 529.1356(M+H-2H_2_O, C_26_H_25_O_12_);511.1238(M+H-3H_2_O, C_26_H_23_O_11_); 427.1021(M+H-H_2_O-C_4_H_8_O_4_,C_22_H_19_O_9_);409.0933(M+H-2H_3_O -C_4_H_8_O_4_,C_22_H_17_O_8_); 391.0817(M+H-3H_2_O-C_4_H_8_O_4_, C_22_H_15_O_7_) | | | | | | | | | | | | | | | | | | | Schaftoside | | | | | | | | | | | | | | F | | | | |
|  | [M-H]^-^ | 12.25 | C_26_H_28_O_14_ | 563.1406 | 563.1411 | 0.9 | | | | | | 563.1431(M-H,C_26_H_27_O_14_);503.1198(M-H-C_2_H_4_O_2_, C_24_H_23_O_12_);473.1102 (M-H-C_3_H_6_O_3_, C_23_H_21_O_11_);443.0990(M-H-C_4_H_8_O_4_, C_22_H_19_O_10_);383.0781 (M-H-C_3_H_6_O_3_-C_3_H_6_O_3_, C_20_H_15_O_8_);353.0678(M-H-C_4_H_8_O_4_-C_3_H_6_O_3_, C_19_H_13_O_7_) | | | | | | | | | | | | | | | | | | |  |  |  |  |  |  |  |  |  |  |  |  |  |  |  |  |  |  |  |
| 31 | [M-H]^-^ | 11.86 | C_20_H_32_O_7_ | 383.2075 | 383.2076 | 0.2 | | | | | | 383.2087(M-H,C_20_H_31_O_7_);381.1920(M-3H,C_20_H_29_O_7_);  365.1989(M-H-H_2_O,C_20_H_29_O_6_) | | | | | | | | | | | | | | | | | | | 3-Deoxyryanodol | | | | | | | | | | | | | | O | | | | |
| 32 | [M-H]^-^ | 12.12 | C_32_H_40_O_19_ | 727.2091 | 727.2097 | 0.9 | | | | | | 727.2093(M-H, C_32_H_39_O_19_);565.1569(M-H-C_6_H_10_O_5_, C_26_H_29_O_14_) | | | | | | | | | | | | | | | | | | | Parishin C | | | | | | | | | | | | | | O | | | | |
| 33 | [M+H]^+^ | 12.22 | C_21_H_20_O_9_ | 417.1180 | 417.1181 | 0.2 | | | | | | 417.1174(M+H, C_21_H_21_O_9_); 255.0664(M+H-C_6_H_10_O_5_, C_15_H_11_O_4_); 227.0770(M+H- C_6_H_10_O_5_-CO, C_14_H_11_O_3_); 199.0770(M+H-C_6_H_10_O_5_-2CO, C_13_H_11_O_2_) | | | | | | | | | | | | | | | | | | | Chrysophanein | | | | | | | | | | | | | | AN | | | | |
| 34 | [M+H]^+^ | 12.22 | C_21_H_20_O_9_ | 417.1180 | 417.1181 | 0.4 | | | | | | 417.1174(M+H, C_21_H_21_O_9_);255.0664(M+H-C_6_H_10_O_5_,C_15_H_11_O_4_) | | | | | | | | | | | | | | | | | | | Daidzin | | | | | | | | | | | | | | F | | | | |
| 35 | [M-H]^-^ | 12.31 | C_27_H_32_O_14_ | 579.1719 | 579.1717 | -0.4 | | | | | | 579.1730(M-H,C_27_H_31_O_14_);255.0668(M-H-C_6_H_10_O_4_-C_6_H_10_O_5_-O,C_15_H_11_O_4_); 135.0089 (M-H-C_6_H_10_O_4_-C_6_H_10_O_5_-C_8_H_8_O-O,C_7_H_3_O_3_);  119.0503(M-H-C_6_H_10_O_4_-C_6_H_10_O_5_-C_7_H_4_O_4_,C_8_H_7_O) | | | | | | | | | | | | | | | | | | | Naringin | | | | | | | | | | | | | | F | | | | |
| 36 | [M-H]^-^ | 12.71 | C_9_H_8_O_2_ | 147.0452 | 147.0453 | 0.9 | | | | | | 147.0458(M-H,C_9_H_7_O_2_);129.0348(M-H-H_2_O,C_9_H_5_O);  119.0500(M-H-CO,C_8_H_7_O);117.0346(M-H-CO-H_2_,C_8_H_5_O) | | | | | | | | | | | | | 2-Hydroxy cinnamaldehyde | | | | | | | | | | | | | | | | | | | | OA | | | | |
| 37 | [M+H]^+^ | 12.99 | C_27_H_32_O_14_ | 581.1865 | 581.1866 | 0.2 | | | | | | 581.1965(M+H, C_27_H_33_O_14_);257.0810(M+H-C_6_H_10_O_4_-C_6_H_10_O_5_-O, C_15_H_13_O_4_) ;137.0229(M+H-C_6_H_10_O_4_-C_6_H_10_O_5_-C_8_H_8_O-O, C_7_H_5_O_3_) | | | | | | | | | | | | | | | | | | | Naringin | | | | | | | | | | | | | | F | | | | |
| 38 | [M+H]^+^ | 13.16 | C_22_H_22_O_10_ | 447.1286 | 447.1283 | -0.6 | | | | | | 447.1289(M+H,C_22_H_23_O_10_);285.0756(M+H-C_6_H_10_O_5_, C_16_H_13_O_5_);  270.0521 (M+H-C_6_H_10_O_5_-CH_3_, C_15_H_10_O_5_) | | | | | | | | | | | | | | | | | | | Calycosin-7-O-glucoside | | | | | | | | | | | | | | F | | | | |
| 39 | [M-H]^-^ | 13.19 | C_15_H_12_O_7_ | 303.0510 | 303.0510 | 0 | | | | | | 285.0498(M-H-H_2_O, C_15_H_9_O_6_); 275.0530 (M-H-CO, C_14_H_11_O_6_); 259.0654(M-H-CO-O, C_14_H_11_O_5_) ; 178.9994(M-H-C_6_H_3_O_2_-H_2_O, C_9_H_6_O_4_); 125.0251(M-H-C_9_H_6_O_4_, C_6_H_5_O_3_) | | | | | | | | | | | | | | | | | | | | | | | Taxifolin | | | | | | | | | | F | | | | |
| 40 | [M+H]^+^ | 13.20 | C_21_H_22_O_9_ | 419.1337 | 419.1333 | -0.8 | | | | | | 419.1342(M+H, C_21_H_23_O_9_);257.0815(M+H-C_6_H_10_O_5_, C_15_H_13_O_4_); 147.0438(M+H -C_6_H_10_O_5_-C_6_H_6_O_2_, C_9_H_7_O_2_) ;137.0235(M+H-C_6_H_10_O_5_-C_8_H_8_O, C_7_H_5_O_3_) | | | | | | | | | | | | | | | | | | | Neoliquiritin | | | | | | | | | | | | | | F | | | | |
|  | [M-H]^-^ | 13.31 | C_21_H_22_O_9_ | 417.1191 | 417.1198 | 1.7 | | | | | | 417.1195(M-H,C_21_H_21_O_9_);255.0681(M-H-C_6_H_10_O_5_,C_15_H_11_O_4_);135.0093 (M-H-C_6_H_10_O_5_-C_8_H_8_O,C_7_H_3_O_3_);119.0511(M-H-C_6_H_10_O_5_-C_7_H_4_O_3_,C_8_H_7_O) | | | | | | | | | | | | | | | | | | |  |  |  |  |  |  |  |  |  |  |  |  |  |  |  |  |  |  |  |
| 41 | [M+H]^+^ | 13.21 | C_15_H_12_O_4_ | 257.0808 | 257.0810 | 0.8 | | | | | | 257.0805(M+H, C_15_H_13_O_4_); 165.0691(M+H-C_6_H_4_O, C_9_H_9_O_3_); 147.0439(M+H-C_6_H_5_O-OH, C_9_H_7_O_2_); 137.0234(M+H-C_8_H_8_O, C_7_H_5_O_3_); 119.0487(M+H-C_8_H_8_O-H_2_O, C_7_H_3_O_2_); 109.0277(M+H-C_8_H_8_O-CO, C_6_H_5_O_2_) | | | | | | | | | | | | | | | | | | | Liquiritigenin^*^ | | | | | | | | | | | | | | F | | | | |
| 42 | [M-H]^-^ | 13.22 | C_11_H_10_O_5_ | 221.0455 | 221.0454 | -0.5 | | | | | | 221.1466(M-H, C_11_H_9_O_5_);162.0340(M-H-CO_2_-CH_3_,C_9_H_6_O_3_) | | | | | | | | | | | | | | | | | | | Isofraxidin | | | | | | | | | | | | | | P | | | | |
| 43 | [M+H]^+^ | 13.44 | C_21_H_22_O_9_ | 419.1337 | 419.1333 | -0.9 | | | | | | 419.1405(M+H, C_21_H_23_O_9_);257.0816(M+H-C_6_H_10_O_5_, C_15_H_13_O_4_); 147.0438 (M+H-C_6_H_10_O_5_-C_6_H_6_O_2_, C_9_H_7_O_2_) ;137.0235(M+H-C_6_H_10_O_5_-C_8_H_8_O, C_7_H_5_O_3_) | | | | | | | | | | | | | | | | | | | Liquiritin^*^ | | | | | | | | | | | | | | F | | | | |
|  | [M-H]^-^ | 13.58 | C_21_H_22_O_9_ | 417.1191 | 417.1194 | 0.7 | | | | | | 417.1217(M-H,C_21_H_21_O_9_);255.0702(M-H-C_6_H_10_O_5_,C_15_H_11_O_4_);148.0173 (M-H-C_6_H_10_O_5_-C_6_H_3_O_2_,C_9_H_8_O_2_);135.0100(M-H-C_6_H_10_O_5_-C_8_H_8_O,C_7_H_3_O_3_); 119.0515(M-H-C_6_H_10_O_5_-C_7_H_4_O_3_,C_8_H_7_O) | | | | | | | | | | | | | | | | | | |  |  |  |  |  |  |  |  |  |  |  |  |  |  |  |  |  |  |  |
| 44 | [M+H]^+^ | 13.47 | C_27_H_30_O_14_ | 579.1708 | 579.1705 | -0.6 | | | | | | 579.1723(M+H, C_27_H_31_O_14_); 561.1614(M+H-H_2_O, C_27_H_29_O_13_); 543.1511 (M+H-2H_2_O, C_27_H_27_O_12_); 525.1408(M+H-3H_2_O, C_27_H_25_O_11_); 495.1294 (M+H-3H_2_O-CH_2_O, C_26_H_23_O_10_); 459.1176(M+H-C_4_H_8_O_4_, C_23_H_23_O_10_); 325.0711 (M+H-7CH_2_O-C_2_H_4_O, C_18_H_13_O_6_); 295.0597(M+H-8CH_2_O-C_2_H_4_O, C_17_H_11_O_5_) | | | | | | | | | | | | | | | | | | | Isoviolanthin | | | | | | | | | | | | | | F | | | | |
| 45 | [M+H]^+^ | 13.46 | C_26_H_30_O_13_ | 551.1759 | 551.1756 | -0.5 | | | | | | 551.1930(M+H, C_26_H_31_O_13_); 257.0814(M+H-C_5_H_8_O_4_-C_6_H_10_O_5_,C_15_H_13_O_4_); 147.0439(M+H-C_5_H_8_O_4_-C_6_H_10_O_5_-C_6_H_6_O_2_,C_9_H_7_O_2_); 137.0230(M+H-C_5_H_8_O_4_-C_6_H_10_O_5_-C_8_H_8_O,C_7_H_5_O_3_) | | | | | | | | | | | | | | | | | | | Liquiritin apioside | | | | | | | | | | | | | | F | | | | |
|  | [M-H]^-^ | 13.80 | C_26_H_30_O_13_ | 549.1614 | 549.1616 | 0.4 | | | | | | 549.1721(M-H,C_26_H_29_O_13_);255.0704(M-H-C_6_H_10_O_5_-C_5_H_8_O_4_,C_15_H_11_O_4_); 135.0106(M-H-C_6_H_10_O_5_-C_5_H_8_O_4_-C_8_H_8_O,C_7_H_3_O_3_);  119.0517(M-H-C_6_H_10_O_5_-C_5_H_8_O_4_-C_7_H_4_O_3_,C_8_H_7_O) | | | | | | | | | | | | | | | | | | |  |  |  |  |  |  |  |  |  |  |  |  |  |  |  |  |  |  |  |
| 46 | [M-H]^-^ | 14.14 | C_10_H_10_O_3_ | 177.0559 | 177.0559 | 0.8 | | | | | | 177.0554(M-H, C_10_H_9_O_3_); 162.0325(M-H-CH_3_, C_9_H_6_O_3_); 117.0339(M-H-CH_3_-COOH, C_8_H_5_O); | | | | | | | | | | | | | | | | | | | 2-Methoxycinnamic acid | | | | | | | | | | | | | | P | | | | |
| 47 | [M-H]^-^ | 14.14 | C_17_H_16_O_6_ | 315.0784 | 315.0876 | 0.6 | | | | | | 315.0874(M-H,C_17_H_15_O_6_);271.1000(M-H-CO-O,C_16_H_15_O_4_);256.0682 (M-H-CO-O-CH_3_,C_15_H_12_O_4_);135.0465(M-H-CO-O-CH_3_-C_7_H_5_O_2_,C_8_H_7_O_2_); 109.0302(M-H-CO-O-CH_3_-C_7_H_5_O_2_-C_2_H_2_,C_6_H_5_O_2_) | | | | | | | | | | | | | | | | | | | 1,2,3,7-Tetramethoxyxanthone | | | | | | | | | | | | | | F | | | | |
| 48 | [M+H]^+^ | 14.28 | C_9_H_6_O_2_ | 147.0441 | 147.0442 | 0.8 | | | | | | 147.0443(M+H, C_9_H_7_O_2_);103.0540(M+H-CO_2_,C_8_H_7_) | | | | | | | | | | | | | | | | | | | Coumarin^*^ | | | | | | | | | | | | | | P | | | | |
| 49 | [M+H]^+^ | 15.11 | C_15_H_10_O_5_ | 271.0601 | 271.0603 | 0 | | | | | | 271.0607(M+H, C_15_H_11_O_5_); 161.0236(M+H-C_6_H_6_O_2_, C_9_H_5_O_3_); 137.0225(M+H-C_8_H_6_O_2_, C_7_H_5_O_3_); 135.0427(M+H-C_7_H_4_O_3_, C_8_H_7_O_2_) | | | | | | | | | | | | | | | | | | | 3',4',7-Trihydroxyflavone | | | | | | | | | | | | | | F | | | | |
|  | [M-H]^-^ | 15.44 | C_15_H_10_O_5_ | 269.0456 | 269.0456 | 0 | | | | | | 269.0469(M-H,C_15_H_9_O_5_);135.0093(M-H-C_8_H_6_O_2_,C_7_H_3_O_3_); 133.0300(M-H-C_7_H_4_O_3_,C_8_H_5_O_2_) | | | | | | | | | | | | | | | | | | |  |  |  |  |  |  |  |  |  |  |  |  |  |  |  |  |  |  |  |
| 50 | [M+H]^+^ | 15.11 | C_15_H_10_O_5_ | 271.0601 | 271.0603 | 0.9 | | | | | | 271.0607(M+H, C_15_H_11_O_5_); 153.0168(M+H-C_8_H_6_O, C_7_H_5_O_4_); 119.0501(M+H-C_7_H_4_O_4_, C_8_H_7_O) | | | | | | | | | | | | | | | | | | | Genistein | | | | | | | | | | | | | | F | | | | |
|  | [M-H]^-^ | 15.44 | C_15_H_10_O_5_ | 269.0460 | 269.0456 | 0 | | | | | | 269.0469(M-H,C_15_H_9_O_5_);133.0300(M-H-C_6_H_4_O_2_-CO,C_8_H_5_O_2_) | | | | | | | | | | | | | | | | | | |  |  |  |  |  |  |  |  |  |  |  |  |  |  |  |  |  |  |  |
| 51 | [M+H]^+^ | 15.16 | C_15_H_10_O_7_ | 303.0499 | 303.0497 | -0.9 | | | | | | 303.0533(M+H, C_15_H_11_O_7_); 285.0398(M+H-H_2_O, C_15_H_9_O_6_); 257.0444(M+H-H_2_O-CO, C_14_H_9_O_5_); 229.0498(M+H-H_2_O-2CO, C_13_H_9_O_4_); 153.0170(M+H-C_8_H_6_O_3_,C_7_H_5_O_4_); 137.0207(M+H-C_8_H_6_O_3_-O,C_7_H_5_O_3_); | | | | | | | | | | | | | | | | | | | Quercetin | | | | | | | | | | | | | | F | | | | |
| 52 | [M-H]^-^ | 15.22 | C_25_H_24_O_12_ | 515.1195 | 515.1191 | -0.9 | | | | | | 515.1194(M-H, C_25_H_23_O_12_); 353.0898(M-H-C_9_H_6_O_3_,C_16_H_17_O_9_); 191.0567 (M-H-2C_9_H_6_O_3_,C_7_H_11_O_6_); 179.0354(M-H-C_16_H_16_O_8_,C_9_H_7_O_4_); 173.0462 (M-H-2C_9_H_6_O_3_-H_2_O,C_7_H_9_O_5_); 135.0463(M-H-C_16_H_16_O_8_-CO_2_,C_8_H_7_O_2_) | | | | | | | | | | | | | | | | | | | Isochlorogenic acid B^*^ | | | | | | | | | | | | | | P | | | | |
| 53 | [M+H]^+^ | 15.36 | C_27_H_28_O_14_ | 577.1552 | 577.1546 | -1 | | | | | | 577.1639(M+H, C_27_H_29_O_14_); 559.1483(M+H-H_2_O, C_27_H_27_O_13_); 313.0705 (M+H-C_11_H_20_O_7_,C_16_H_9_O_7_); 295.0610(M+H-C_11_H_20_O_7_-H_2_O, C_16_H_7_O_6_) | | | | | | | | | | | | | | | | | | | Cassiaoccidentalin B | | | | | | | | | | | | | | F | | | | |
| 54 | [M-H]^-^ | 15.41 | C_25_H_24_O_12_ | 515.1195 | 515.1188 | -1.3 | | | | | | 515.1771(M-H, C_25_H_23_O_12_); 353.0885(M-H-C_9_H_6_O_3_,C_16_H_17_O_9_); 191.0560 (M-H-2C_9_H_6_O_3_,C_7_H_11_O_6_); 179.0359(M-H-C_16_H_16_O_8_,C_9_H_7_O_4_); 135.0452(M-H-C_16_H_16_O_8_-CO_2_,C_8_H_7_O_2_) | | | | | | | | | | | | | | | | | | | Isochlorogenic acid A^*^ | | | | | | | | | | | | | | P | | | | |
| 55 | [M+H]^+^ | 15.57 | C_22_H_22_O_11_ | 463.1235 | 463.1237 | 0.4 | | | | | | 463.1182(M+H, C_22_H_23_O_11_);301.0709(M+H-C_6_H_10_O_5_, C_16_H_13_O_6_) ;286.0456(M+H-C_6_H_10_O_5_-CH_3_, C_15_H_10_O_6_); | | | | | | | | | | | | | | | | | | | Tectoridin | | | | | | | | | | | | | | F | | | | |
| 56 | [M-H]^-^ | 15.65 | C_15_H_10_O_5_ | 269.0456 | 269.0456 | 0.2 | | | | | | 269.0462(M-H, C_15_H_9_O_5_);241.0500(M-H-CO, C_14_H_9_O_4_);213.0562(M-H-2CO,C_13_H_9_O_3_) | | | | | | | | | | | | | | | | | | | | | | | | | | | | | | Emodin | | | AN | | | | |
| 57 | [M-H]^-^ | 15.66 | C_16_H_12_O_6_ | 299.0561 | 299.0564 | 1 | | | | | | 299.0577(M-H,C_16_H_11_O_6_);240.0441(M-H-CH_3_O-CO, C_14_H_8_O_4_); 239.0361(M-H-CH_3_O-CO-H, C_14_H_7_O_4_);212.0488(M-H-CH_3_O-2CO, C_13_H_8_O_3_); | | | | | | | | | | | | | | | | | | | Xanthorin | | | | | | | | | | | | | | AN | | | | |
| 58 | [M-H]^-^ | 15.87 | C_16_H_14_O_6_ | 301.0718 | 301.0720 | 0.9 | | | | | | 301.0723(M-H,C_16_H_13_O_6_);286.0491(M-H-CH_3_, C_15_H_10_O_6_);  191.0351 (M-H-C_6_H_6_O_2_,C_10_H_7_O_4_);161.0253(M-H-C_7_H_7_O_3_-H,C_9_H_5_O_3_);  150.0325(M-H-CH_3_-C_7_H_4_O_3_,C_8_H_6_O_3_) | | | | | | | | | | | | | | | | | | | 3,4,3',4'-Tetrahydroxy-2-methoxychalcone | | | | | | | | | | | | | | F | | | | |
| 59 | [M-H]^-^ | 15.94 | C_21_H_22_O_10_ | 433.1140 | 433.1138 | -0.4 | | | | | | 433.1161(M-H,C_21_H_21_O_10_);271.0637(M-H-C_6_H_10_O_5_, C_15_H_11_O_5_); 151.0048(M-H-C_6_H_10_O_5_-C_8_H_8_O,C_7_H_3_O_4_);119.0511(M-H-C_6_H_10_O_5_-C_7_H_4_O_4_,C_8_H_7_O) | | | | | | | | | | | | | | | | | | | Isosalipurposide | | | | | | | | | | | | | | F | | | | |
| 60 | [M-H]^-^ | 15.98 | C_17_H_14_O_6_ | 313.0718 | 313.0716 | -0.7 | | | | | | 313.0722(M-H,C_17_H_13_O_6_);298.0499(M-H-CH_3_,C_16_H_10_O_6_);269.0838(M-H-CH_3_ -CHO,C_15_H_9_O_5_);253.0510(M-H-CH_3_-CHO-O,C_15_H_9_O_4_);237.0541(M-H-CH_3_ -CHO-2O,C_15_H_9_O_3_);225.0558(M-H-CH_3_-CHO-O-CO,C_14_H_9_O_3_) | | | | | | | | | | | | | | | | | | | Kumatakenin | | | | | | | | | | | | | | F | | | | |
|  | [M+H]^+^ | 19.72 | C_17_H_14_O_6_ | 315.0863 | 315.0863 | 0 | | | | | | 315.0867(M+H, C_17_H_15_O_6_);300.0655 (M+H-CH_3_,C_16_H_12_O_6_); 167.0351(M+H-C_9_H_8_O_2_, C_8_H_7_O_4_);139.0517 (M+H-C_9_H_8_O_2_-CO,C_7_H_7_O_3_) | | | | | | | | | | | | | | | | | | |  |  |  |  |  |  |  |  |  |  |  |  |  |  |  |  |  |  |  |
| 61 | [M-H]^-^ | 16.39 | C_20_H_30_O_6_ | 365.1970 | 365.1969 | -0.1 | | | | | | 365.1980(M-H,C_20_H_29_O_6_);285.1862(M-H-CO_2_-2H_2_O,C_19_H_25_O_2_); 267.1785(M-H-CO_2_-3H_2_O,C_19_H_23_O);167.1086(M-H-CO_2_-C_9_H_14_O_2_, C_10_H_15_O_2_);137.0953(M-H-CO_2_-C_9_H_14_O_2_-2CH_3_,C_8_H_9_O_2_) | (3aR)-3a,4,5,6,7,7a,8,8a-Octahydro-3aβ,4α,7aβ,8aβ-tetrahydroxy-3,5β,8-trimethyl-2-isopropyl-1H-3bα,8α-(epoxyethano)cyclopent[a]inden-10-one | | | | | | | | | | | | | | | | | | | | | | | | | | | | | | | | | | | | L |
| 62 | [M-H]^-^ | 16.46 | C_25_H_24_O_14_ | 515.1195 | 547.1079 | -0.9 | | | | | | 515.1178(M-H, C_25_H_23_O_12)_; 353.0894(M-H-C_9_H_6_O_3_,C_16_H_17_O_9_); 191.0566 (M-H-2C_9_H_6_O_3_,C_7_H_11_O_6_); 179.0356(M-H-C_16_H_16_O_8_,C_9_H_7_O_4_); 173.0458 (M-H-2C_9_H_6_O_3_-H_2_O,C_7_H_9_O_5_); 135.0452(M-H-C_16_H_16_O_8_-CO_2_,C_8_H_7_O_2_) | | | | | | | | | | | | | | | | | | | Isochlorogenic acid C^*^ | | | | | | | | | | | | | | P | | | | |
| 63 | [M+H]^+^ | 16.48 | C_20_H_22_O_4_ | 327.1591 | 327.1591 | -0.1 | | | | | | 327.1643(M+H,C_20_H_23_O_4_);163.0743(M+H-C_10_H_12_O_2_,C_10_H_11_O_2_); 137.0594(M+H-C_12_H_14_O_2_,C_8_H_9_O_2_) | | | | | | | | | | | | | | | | | | | Dehydrodiisoeugenol | | | | | | | | | | | | | | P | | | | |
| 64 | [M-H]^-^ | 16.81 | C_20_H_26_O_6_ | 361.4657 | 361.1656 | -0.3 | | | | | | 361.1683(M-H,C_20_H_25_O_6_);346.1406(M-H-CH_3_,C_19_H_22_O_6_);179.0712(M-H -C_10_H_14_O_3_,C_10_H_11_O_3_);165.0559(M-H-C_10_H_13_O_3_-CH_3_,C_9_H_9_O_3_);147.0456(M-H -C_10_H_13_O_3_-CH_3_-H_2_O,C_9_H_7_O_2_);121.0292(M-H-C_10_H_13_O_3_-CH_3_-H_2_O-C_2_H_2_,C_7_H_5_O_2_) | | | | | | | | | | | | | | | | | | | Secoisolariciresinol | | | | | | | | | | | | | | P | | | | |
| 65 | [M+H]^+^ | 17.03 | C_15_H_10_O_4_ | 255.0652 | 255.0654 | 0.9 | | | | | | 255.0651(M+H, C_15_H_11_O_4_); 145.0274(M+H-C_6_H_6_O_2_, C_9_H_5_O_2_); 137.0226(M+H-C_8_H_6_O, C_7_H_5_O_3_) | | | | | | | | | | | | | | | | | | | 7,4'-Dihydroxyflavone | | | | | | | | | | | | | | F | | | | |
|  | [M-H]^-^ | 17.35 | C_15_H_10_O_4_ | 253.0509 | 253.0509 | 1 | | | | | | 253.0517(M-H, C_15_H_9_O_4_); 135.0097(M-H-C_8_H_6_O,C_7_H_3_O_3_); 117.0350(M-H-C_7_H_4_O_3_,C_8_H_5_O) | | | | | | | | | | | | | | | | | | |  |  |  |  |  |  |  |  |  |  |  |  |  |  |  |  |  |  |  |
| 66 | [M+H]^+^ | 17.13 | C_26_H_30_O_13_ | 551.1759 | 551.1763 | 0.9 | | | | | | 551.1752(M+H, C_26_H_31_O_13_); 257.0816(M+H-C_5_H_8_O_4_-C_6_H_10_O_5_,C_15_H_13_O_4_); 147.0440(M+H-C_5_H_8_O_4_-C_6_H_10_O_5_-C_6_H_6_O_2_,C_9_H_7_O_2_); 137.0230(M+H-C_5_H_8_O_4_-C_6_H_10_O_5_-C_8_H_8_O,C_7_H_5_O_3_) | | | | | | | | | | | | | | | | | | | | | | | | | Isoliquiritin apioside^*^ | | | | | | | | F | | | | |
|  | [M-H]^-^ | 17.44 | C_26_H_30_O_13_ | 549.1614 | 549.1618 | 0.7 | | | | 549.1635(M-H,C_26_H_29_O_13_);255.0675(M-H-C_6_H_10_O_5_-C_5_H_8_O_4_,C_15_H_11_O_4_);135.0097(M -H-C_6_H_10_O_5_-C_5_H_8_O_4_-C_8_H_8_O,C_7_H_3_O_3_);119.0509(M-H-C_6_H_10_O_5_-C_5_H_8_O_4_-C_7_H_4_O_3_,C_8_H_7_O) | | | | | | | | | | | | | | | | | | | | | | | | | | |  |  |  |  |  |  |  |  |  |  |  |  |  |
| 67 | [M+H]+ | 17.13 | C_21_H_22_O_9_ | 419.1337 | 419.1339 | 0.7 | | | | | | 419.1329(M+H, C_21_H_23_O_9_);257.0811(M+H-C_6_H_10_O_5_, C_15_H_13_O_4_); 147.0441 (M+H-C_6_H_10_O_5_-C_6_H_6_O_2_, C_9_H_7_O_2_) ; 137.0232(M+H-C_6_H_10_O_5_-C_8_H_8_O, C_7_H_5_O_3_) | | | | | | | | | | | | | | | | | Neoisoliquiritin | | | | | | | | | | | | | | | | F | | | | |
| 68 | [M-H]^-^ | 17.14 | C_17_H_16_O_5_ | 299.0925 | 299.0925 | 0 | | | | | | 299.1533(M-H, C_17_H_15_O_5_); 255.1022(M-H-CO-O,C_16_H_15_O_3_); 240.0792(M-H-CO-O-CH_3_, C_15_H_12_O_3_);239.0721(M-H-CO-O-CH_3_-H,C_15_H_11_O_3_);119.0496(M-H-C_9_H_8_O_4_,C_8_H_7_O) | | | | | | | | | | | | | | | | | | | | | | | | | | | | | | | Farrerol | | F | | | | |
| 69 | [M+H]^+^ | 17.32 | C_27_H_30_O_13_ | 563.1759 | 563.1760 | 0.1 | | | | | | 563.2794(M+H,C_27_H_31_O_13_);269.0821(M+H-C_5_H_8_O_4_-C_6_H_10_O_5_, C_16_H_13_O_4_) | | | | | | | | | | | | | | | | | | | Glycyroside | | | | | | | | | | | | | | F | | | | |
| 70 | [M+H]^+^ | 17.35 | C_17_H_18_O_6_ | 319.1176 | 319.1179 | 0.8 | | | | | | 319.1167(M+H, C_17_H_19_O_6_); 153.0543(M+H-C_8_H_10_-2CH_2_O, C_7_H_5_O_4_); 137.0589  (M+H-C_7_H_8_-3CH_2_O,C_7_H_5_O_3_); 123.0433(M+H-C_8_H_10_-3CH_2_O,C_6_H_3_O_3_) | | | | | | | | | | | | | | | | | | | Agarotetrol | | | | | | | | | | | | | | F | | | | |
| 71 | [M+H]^+^ | 17.49 | C_21_H_22_O_9_ | 419.1337 | 419.1338 | 0.4 | | | | | | 419.1349(M+H, C_21_H_23_O_9_);257.0818(M+H-C_6_H_10_O_5_, C_15_H_13_O_4_); 147.0438 (M+H-C_6_H_10_O_5_-C_6_H_6_O_2_, C_9_H_7_O_2_) ;137.0235(M+H-C_6_H_10_O_5_-C_8_H_8_O, C_7_H_5_O_3_) | | | | | | | | | | | | | | | | | | | Isoliquiritin^*^ | | | | | | | | | | | | | | F | | | | |
|  | [M-H]^-^ | 17.79 | C_21_H_22_O_9_ | 417.1191 | 417.1194 | 0.6 | | | | | | 417.1210(M-H,C_21_H_21_O_9_);255.0679(M-H-C_6_H_10_O_5_,C_15_H_11_O_4_);148.0177 (M-H-C_6_H_10_O_5_-C_6_H_3_O_2_,C_9_H_8_O_2_);135.0096(M-H-C_6_H_10_O_5_-C_8_H_8_O,C_7_H_3_O_3_);  119.0509(M-H-C_6_H_10_O_5_-C_7_H_4_O_3_,C_8_H_7_O) | | | | | | | | | | | | | | | | | | |  |  |  |  |  |  |  |  |  |  |  |  |  |  |  |  |  |  |  |
| 72 | [M+H]^+^ | 17.67 | C_22_H_22_O_9_ | 431.1337 | 431.1336 | -0.1 | | | | | | 431.1348(M+H, C_22_H_23_O_9_); 269.0825(M+H-C_6_H_10_O_5_, C_16_H_13_O_4_); 254.0582(M+H-C_6_H_10_O_5_-CH_3_, C_15_H_10_O_4_) | | | | | | | | | | | | | | | | | | | Ononin | | | | | | | | | | | | | | F | | | | |
| 73 | [M+H]^+^ | 17.83 | C_16_H_14_O_5_ | 287.0914 | 287.0916 | 0.7 | | | | | | 287.0960(M+H,C_16_H_15_O_5_ ); 193.0492(M+H-C_6_H_6_O, C_10_H_9_O_4_); 150.0308( M+H-C_7_H_6_O_2_-CH_3_, C_8_H_6_O_3_); 121.0283(M+H-C_9_H_10_O_3_, C_7_H_5_O_2_) | | | | | | | | | | | | | | | | | | | Licochalcone B | | | | | | | | | | | | | | F | | | | |
|  | [M-H]^-^ | 18.14 | C_16_H_14_O_5_ | 285.0769 | 285.0769 | 0.1 | | | | | | 285.0769(M-H, C_16_H_13_O_5_);270.0542(M-H-CH_3_,C_15_H_10_O_5_);  150.0329(M-H-CH_3_ -C_7_H_4_O_2_,C_8_H_6_O_3_);149.0248 (M-H-CH_3_ -C_7_H_4_O_2_-H,C_8_H_5_O_3_);121.0297(M-H-CH_3_-C_8_H_5_O_3_,C_7_H_5_O_2_) | | | | | | | | | | | | | | | | | | |  |  |  |  |  |  |  |  |  |  |  |  |  |  |  |  |  |  |  |
| 74 | [M+H]^+^ | 17.88 | C_15_H_12_O_4_ | 257.0808 | 257.0813 | 1.3 | | | | | | 257.0811(M+H, C_15_H_13_O_4_); 165.0702(M+H-C_6_H_4_O, C_9_H_9_O_3_); 147.0438(M+H-C_6_H_5_O-OH, C_9_H_7_O_2_); 137.0236(M+H-C_8_H_8_O, C_7_H_5_O_3_); 119.0492(M+H-C_8_H_8_O-H_2_O, C_7_H_3_O_2_); 109.0280(M+H-C_8_H_8_O-CO, C_6_H_5_O_2_) | | | | | | | | | | | | | | | | | | | Isoliquiritigenin | | | | | | | | | | | | | | F | | | | |
| 75 | [M-H]^-^ | 18.19 | C_15_H_12_O_5_ | 271.0614 | 271.0612 | 0.8 | | | | | | 271.0615(M-H,C_15_H_11_O_5_); 253.500(M-H-H_2_O,C_15_H_9_O_4_); 243.0675(M-H-CO,C_14_H_11_O_4_) | | | | | | | | | | | | | | | | | | | Toralactone | | | | | | | | | | | | | | P | | | | |
| 76 | [M-H]^-^ | 18.39 | C_15_H_10_O_6_ | 285.0405 | 285.0402 | -0.9 | | | | | | 285.0410(M-H,C_15_H_9_O_6_);257.0469(M-H-CO,C_14_H_9_O_5_);177.0212 (M-H-C_6_H_4_O_2_, C_9_H_5_O_4_);150.0309(M-H-C_8_H_7_O_2_,C_7_H_2_O_4_);133.0314(M-H-C_7_H_4_O_4_,C_8_H_5_O_2_) | | | | | | | | | | | | | | | | | | | Kaempferol | | | | | | | | | | | | | | F | | | | |
| 77 | [M+H]^+^ | 18.61 | C_12_H_16_O_3_ | 209.1172 | 209.1173 | 0.5 | | | | | | 209.1075(M+H, C_12_H_17_O_3_);167.1064(M+H-C_3_H_6_, C_9_H_11_O_3_); 149.0961(M+H-2CH_2_O, C_10_H_13_O);107.0849(M+H-2CH_2_O-C_3_H_6_, C_7_H_7_O) | | | | | | | | | | | | | | | | | | | α-Asarone | | | | | | | | | | | | | | P | | | | |
| 78 | [M+H]^+^ | 19.06 | C_16_H_12_O_5_ | 285.0758 | 285.0760 | 0 | | | | | | 285.0751(M+H, C_16_H_13_O_5_); 270.0514(M+H-CH_3_, C_15_H_10_O_5_); 253.0496(M+H-CH_4_O, C_15_H_9_O_4_); 225.0544(M+H-CH_4_O-CO, C_14_H_9_O_3_); 197.0586(M+H-CH_4_O-2CO, C_13_H_9_O_2_) | | | | | | | | | | | | | | | | | | | | | | | | Physcion | | | | | | | | | AN | | | | |
| 79 | [M-H]^-^ | 19.31 | C_9_H_8_O_2_ | 147.0452 | 147.0453 | 0.9 | | | | | | 147.0411(M-H, C_9_H_7_O_2_);103.0549(M-H-CO_2_,C_8_H_7_) | | | | | | | Cinnamic acid^*^ | | | | | | | | | | | | | | | | | | | | | | | | | | OA | | | | |
| 80 | [M+H]^+^ | 19.67 | C_16_H_16_O_5_ | 289.1071 | 289.1070 | -0.1 | | | | | | 187.0737(M+H-C_4_H_6_O_3_,C_12_H_11_O_2_) ;161.0590(M+H-C_6_H_8_O_3_,C_10_H_9_O_2_); 118.0401(M+H-C_8_H_11_O_4_,C_8_H_6_O) | | | | | | | | | | | | | | | | | | | Columbianetin acetate | | | | | | | | | | | | | | P | | | | |
| 81 | [M-H]^-^ | 21.26 | C_15_H_12_O_5_ | 271.0614 | 271.0614 | 0.8 | | | | | | 271.0615(M-H,C_15_H_11_O_5_);151.0040(M-H-C_8_H_8_O,C_7_H_3_O_4_); 119.0508(M-H-C_7_H_4_O_4_,C_8_H_7_O);107.0141(M-H-C_8_H_8_O-CO-O,C_6_H_3_O_2_) | | | | | | | | | | | | | | | | | | | Naringenin | | | | | | | | | | | | | | F | | | | |
| 82 | [M-H]^-^ | 21.27 | C_42_H_66_O_16_ | 825.4278 | 825.4281 | 0.3 | | | | | | 825.4324(M-H, C_42_H_65_O_16_);351.0574(M-H-C_6_H_10_O_5_ -C_5_H_8_O_4_-C_2_H_2_O_2_-CO_2_-H_2_O-CH_2_O-2CH_3_, C_25_H_35_O) | | | | | | | | | | | | | | | | | | | Esculentoside A | | | | | | | | | | | | | | T | | | | |
| 83 | [M-H]^-^ | 21.60 | C_16_H_14_O_4_ | 269.0822 | 269.0822 | 1 | | | | | | 269.0825(M-H,C_16_H_13_O_4_);237.0569(M-H-CH_4_O,C_15_H_9_O_3_) ; 209.0609  (M-H-CH_4_O-CO,C_14_H_9_O_2_) ;175.0403(M-H-C_6_H_6_O,C_10_H_7_O_3_);161.0248  (M-H-C_6_H_5_O-CH_3_,C_9_H_5_O_3_); 133.0295(M-H-C_7_H_5_O_2_-CH_3_,C_8_H_5_O_2_) ; 120.0216(M-H-C_9_H_9_O_2_,C_7_H_4_O_2_);108.0207(M-H-C_9_H_6_O_2_-CH_3_,C_6_H_4_O_2_) | | | | | | | | | | | | | | | | | | | Retrochalcone | | | | | | | | | | | | | | F | | | | |
| 84 | [M+H]^+^ | 21.77 | C_16_H_12_O_5_ | 285.0758 | 285.0760 | 0.9 | | | | | | 285.0784(M+H, C_16_H_13_O_5_); 225.0555(M+H-CH_3_-OH-CO, C_14_H_9_O_3_); 137.0231(M+H-C_9_H_8_O_2_,C_7_H_5_O_3_) | | | | | | | | | | | | | | | | | | | Calycosin | | | | | | | | | | | | | | F | | | | |
| 85 | [M+H]^+^ | 24.91 | C_16_H_12_O_4_ | 269.0808 | 269.0810 | 0.8 | | | | | | 269.0815(M+H, C_16_H_13_O_4_ ); 253.0503(M+H-O, C_16_H_13_O_3_);  237.0547(M+H -CH_4_O, C_15_H_9_O_3_); 225.0544(M+H-O-CO, C_15_H_13_O_2_); 118.0409(M+H-C_7_H_4_O_3_-CH_3_, C_8_H_6_O) | | | | | | | | | | | | | | | | | | | Formononetin | | | | | | | | | | | | | | F | | | | |
|  | [M-H]^-^ | 25.47 | C_16_H_12_O_4_ | 267.0665 | 267.0668 | 0.7 | | | | | | 267.0664(M-H,C_16_H_11_O_4_); 252.0430(M-H-CH_3_,C_15_H_8_O_4_); 251.0352(M-H-CH_3_-H,C_15_H_7_O_4_); 223.0399(M-H-CH_3_-H-CO,C_14_H_7_O_3_); 135.0085(M-H-C_9_H_8_O,C_7_H_3_O_3_); 132.0210(M-H-C_7_H_4_O_3_,C_9_H_7_O) | | | | | | | | | | | | | | | | | | |  |  |  |  |  |  |  |  |  |  |  |  |  |  |  |  |  |  |  |
| 86 | [M-H]^-^ | 25.10 | C_30_H_46_O_6_ | 501.3222 | 501.3213 | -1.2 | | | | | | 501.3234(M-H,C_30_H_45_O_6_);457.3310(M-H-CO_2_,C_29_H_45_O_4_);441.3107 (M-H-C_2_H_4_O_2_,C_28_H_41_O_4_);403.2496(M-H-C_6_H_10_O,C_24_H_35_O_5_);  385.2360(M-H-C_6_H_10_O-H_2_O,C_24_H_33_O_4_) | | | | | | | | | | | | | | | | | | | 26-Hydroxyporicoic acid G | | | | | | | | | | | | | | T | | | | |
| 87 | [M-H]^-^ | 26.23 | C_16_H_16_O_4_ | 271.0976 | 271.0976 | 0.2 | | 271.1000(M-H,C_16_H_15_O_4_);256.0719(M-H-CH_3_,C_15_H_12_O_4_);241.0512(M-H-CH_2_O,C_15_H_13_O_3_);149.0600(M-H-C_7_H_6_O_2_,C_9_H_9_O_2_);135.0447(M-H-CH_2_O-C_6_H_2_O_2_, C_9_H_11_O);121.0296(M-H-C_9_H_10_O_2_,C_7_H_5_O_2_);109.0305(M-H-CH_2_O-C_9_H_8_O,C_6_H_5_O_2_) | | | | | | | | | | | | | | | | | | | | | | | 7,4'-Dihydroxy-3'-methoxyisoflavan | | | | | | | | | | | | | | F | | | | |
| 88 | [M+H]^+^ | 28.84 | C_42_H_62_O_17_ | 839.4060 | 839.4071 | 1 | | | | | | 839.4108(M+H, C_42_H_63_O_17_); 487.3431(M+H-2C_6_H_8_O_6_, C_30_H_47_O_5_); 469.3325 (M+H-2C_6_H_8_O_6_-H_2_O, C_30_H_45_O_4_); 451.3213(M+H-2C_6_H_8_O_6_-2H_2_O, C_30_H_43_O_3_) | | | | | | | | | | | | | | | | | | | Licoricesaponin G2 | | | | | | | | | | | | | | T | | | | |
| 89 | [M+H]^+^ | 29.53 | C_32_H_48_O_5_ | 513.3575 | 513.3570 | -1 | | | | | | 513.3575(M+H, C_32_H_49_O_5_); 495.3490(M+H-H_2_O, C_32_H_47_O_4_); 453.3337(M+H-C_2_H_4_O_2_, C_30_H_45_O_3_) | | | | | | | | | | | | | | | | | | | Poricoic acid AM | | | | | | | | | | | | | | T | | | | |
| 90 | [M+H]^+^ | 30.62 | C_42_H_62_O_17_ | 839.4060 | 839.4068 | 1 | | | | | | 839.4079(M+H, C_42_H_63_O_17_); 487.3414(M+H-2C_6_H_8_O_6_, C_30_H_47_O_5_); 469.3308 (M+H-2C_6_H_8_O_6_-H_2_O, C_30_H_45_O_4_); 451.3499(M+H-2C_6_H_8_O_6_-2H_2_O, C_30_H_43_O_3_) | | | | | | | | | | | | | | | | | | | Uralsaponin N | | | | | | | | | | | | | | T | | | | |
| 91 | [M+H]^+^ | 30.79 | C_42_H_62_O_16_ | 823.4111 | 823.4117 | 0.7 | | | | | | 823.4148(M+H, C_42_H_63_O_16_); 647.3825(M+H-C_6_H_8_O_6_, C_36_H_55_O_10_); 471.3493 (M+H-2C_6_H_8_O_6_, C_30_H_47_O_4_); 453.3386(M+H-2C_6_H_8_O_6_-H_2_O, C_30_H_45_O_3_) | | | | | | | | | | | | | | | | | | | Glycyrrhizic acid^*^ | | | | | | | | | | | | | | T | | | | |
| 92 | [M+H]^+^ | 30.80 | C_36_H_54_O_10_ | 647.3790 | 647.3791 | 0.2 | | | | | | 647.3816 (M+H, C_36_H_55_O_10_);471.3486(M+H-C_6_H_8_O_6_, C_30_H_47_O_4_); 453.3402(M+H-C_6_H_8_O_6_-H_2_O, C_30_H_45_O_3_) ;435.3273(M+H-C_6_H_8_O_6_-2H_2_O, C_30_H_43_O_2_); 407.3320(M+H-C_6_H_8_O_6_-2H_2_O-CO, C_29_H_43_O) | | | | | | | | | | | | | | | | | | | Glycyrrhetinic acid 3-O-glucuronide | | | | | | | | | | | | | | | T | | | |
| 93 | [M-H]^-^ | 33.83 | C_20_H_18_O_4_ | 321.1132 | 321.1129 | -0.9 | | | | | | 321.1142(M-H,C_20_H_17_O_4_);306.0906(M-H-CH_3_,C_19_H_14_O_4_);305.0833(M-H-O, C_20_H_17_O_3_);291.0666(M-H-2CH_3_,C_18_H_11_O_4_);199.0793(M-H-C_7_H_6_O_2_,C_13_H_11_O_2_); 175.0775(M-H-C_9_H_6_O_2_,C_11_H_11_O_2_);145.0266(M-H-C_11_H_12_O_2_,C_9_H_5_O_2_); 107.0501(M-H-C_9_H_6_O_2_-C_4_H_4_O,C_7_H_7_O) | | | | | | | | | | | | | | | | | | | Glabrene | | | | | | | | | | | | | | F | | | | |
| 94 | [M+H]^+^ | 32.00 | C_21_H_22_O_5_ | 355.1537 | 355.1537 | -1 | | | | | | 355.1557(M+H, C_21_H_23_O_5_); 299.0914(M+H-C_4_H_8_, C_17_H_15_O_5_); 193.0496(M+H-C_11_H_14_O, C_10_H_9_O_4_); 133.0282(M+H-C_8_H_8_O_3_-C_5_H_10_, C_8_H_5_O_2_) | | | | | | | | | | | | | | Licobenzofuran | | | | | | | | | | | | | | | | | | | P | | | | |
| 95 | [M-H]^-^ | 32.30 | C_15_H_22_O_3_ | 249.1496 | 249.1497 | 0.4 | | | | | | 249.1504(M-H,C_15_H_21_O_3_);205.1605(M-H-CO-O,C_14_H_21_O);  203.1450(M-H-2CH_3_-O,C_13_H_15_O_2_);189.1266(M-H-CO-2O,C_14_H_21_);  187.1134(M-H-CH_3_-2O,C_13_H_15_O) | | | | | | | | | | | | | | Nardosinone | | | | | | | | | | | | | | | | | | | O | | | | |
| 96 | [M-H]^-^ | 32.71 | C_15_H_20_O_3_ | 247.1338 | 247.1338 | -0.7 | | | | | | 247.1326(M-H,C_15_H_19_O_3_); 229.1215(M-H-H_2_O,C_15_H_17_O_2_); 203.1450(M-H-CO_2_,C_14_H_19_O) ,201.1287(M-H-H_2_O-CO,C_14_H_17_O) | | | | | | | | | | | | | | Atractylenolide Ⅲ^*^ | | | | | | | | | | | | | | | | | | | L | | | | |
| 97 | [M+H]^+^ | 32.78 | C_20_H_20_O_4_ | 325.1434 | 325.1437 | 0.8 | | | | | | 325.1401(M+H, C_20_H_21_O_4_); 269.0845(M+H-C_4_H_8_,C_16_H_13_O_4_ ); 137.0239(M+H-C_5_H_9_-C_8_H_7_O, C_7_H_5_O_3_) | | | | | | | | | | | | | | Isobavachalcone | | | | | | | | | | | | | | | | | | | F | | | | |
|  | [M-H]^-^ | 33.08 | C_20_H_20_O_4_ | 323.1289 | 323.1287 | -0.8 | | | | | | 323.1294(M-H,C_20_H_19_O_4_);187.1132(M-H-C_6_H_5_-CO_2_-CH_3_,C_12_H_11_O_2_); 135.0092(M-H-C_8_H_8_-C_4_H_7_-CO-H,C_7_H_3_O_3_); 132.0592(M-H-C_6_H_5_-CO_2_-CH_3_-C_4_H_7_,C_8_H_4_O_2_) | | | | | | | | | | | | | |  |  |  |  |  |  |  |  |  |  |  |  |  |  |  |  |  |  |  |  |  |  |  |  |
| 98 | [M+H]^+^ | 33.72 | C_21_H_24_O_5_ | 357.1697 | 357.1676 | 0.8 | | | | | | 357.1609(M+H, C_21_H_25_O_5_); 301.1083(M+H-C_4_H_8_, C_17_H_17_O_5_); 179.0698(M+H-C_6_H_5_O_2_-C_5_H_9_, C_10_H_11_O_3_);165.0544(M+H -C_7_H_6_O_2_-C_5_H_10_,  C_9_H_9_O_3_); 123.0441(M+H-C_9_H_9_O_3_-C_5_H_9_, C_7_H_7_O_2_) | | | | | | | | | | | | | | | | | | | | | | | | | | | Glyasperin C | | | | | | P | | | | |
|  | [M-H]^-^ | 33.99 | C_21_H_24_O_5_ | 355.1551 | 355.1549 | -0.5 | | | | | 355.1546(M-H,C_21_H_23_O_5_);323.1310(M-H-CH_4_O,C_20_H_19_O_4_);233.1169(M-H-C_7_H_6_O_2_,  C_14_H_17_O_3_);175.0400(M-H-C_5_H_10_-C_6_H_6_O_2_,C_10_H_7_O_3_);163.0393(M-H-C_12_H_16_O_2_,C_9_H_7_O_3_);135.0453(M-H-C_13_H_16_O_3_,C_8_H_7_O_2_);109.0281(M-H-C_13_H_16_O_3_-C_2_H_2_,C_6_H_5_O_2_) | | | | | | | | | | | | | | | | | | | | | | | | | | | |  |  |  |  |  |  |  |  |  |  |  |
| 99 | [M+H]^+^ | 36.83 | C_20_H_20_O_4_ | 325.1434 | 325.1437 | 0.7 | | | | | | 325.1458(M+H, C_20_H_21_O_4_); 189.0906(M+H-C_8_H_8_O_2_, C_12_H_13_O_2_); 149.0586 (M+H-C_11_H_10_O-H_2_O, C_9_H_9_O_2_); 123.0437(M+H-C_12_H_12_O_2_-CH_2_, C_7_H_7_O_2_) | | | | | | | | | | | | | | | | | | | Glabridin | | | | | | | | | | | | | | F | | | | |
|  | [M-H]^-^ | 37.08 | C_20_H_20_O_4_ | 323.1289 | 323.1291 | 0.4 | | | | | | 323.1295(M-H,C_20_H_19_O_4_);201.0919(M-H-C_6_H_4_O_2_-CH_2_,C_13_H_13_O_2_);135.0445 (M-H-C_12_H_12_O_2_,C_8_H_7_O_2_);109.0302(M-H-C_12_H_12_O_2_-C_2_H_2_,C_6_H_5_O_2_);107.0506 (M-H-C_12_H_12_O_2_-C_2_H_2_-2H,C_6_H_3_O_2_) | | | | | | | | | | | | | | | | | | |  |  |  |  |  |  |  |  |  |  |  |  |  |  |  |  |  |  |  |
| 100 | [M-H]^-^ | 32.94 | C_20_H_20_O_5_ | 339.1238 | 339.1236 | -0.7 | | | | | | 339.2029(M-H,C_20_H_19_O_5_);245.0833(M-H-C_6_H_6_O,C_14_H_13_O_4_);201.0917(M-H -C_6_H_6_O-CO-O,C_13_H_13_O_2_);177.0899(M-H-C_9_H_6_O_3_,C_11_H_13_O_2_) | | | | | | | | | | | | | | | | | | | 8-Prenylnaringenin | | | | | | | | | | | | | | F | | | | |
| 101 | [M+H]^+^ | 32.97 | C_20_H_20_O_6_ | 357.1333 | 357.1334 | 0.5 | | | | | | 357.1325(M+H, C_20_H_21_O_6_);301.0710(M+H-C_4_H_8_, C_16_H_13_O_6_); 283.0602(M+H-C_4_H_8_-H_2_O, C_16_H_11_O_5_);175.0388(M+H-C_4_H_8_-H_2_O-C_6_H_4_O_2_, C_10_H_7_O_3_);147.0437(M+H-C_4_H_8_-H_2_O-C_6_H_4_O_2_-CO, C_9_H_7_O_2_) | | | | | | | | | | | | | | | | | | | Sigmoidin B | | | | | | | | | | | | | | F | | | | |
|  | [M-H]^-^ | 33.27 | C_20_H_20_O_6_ | 355.1187 | 355.1189 | 0.5 | | | | | | 355.1090(M-H, C_20_H_19_O_6_);229.0883(M-H-C_6_H_4_O_3_-2H,C_14_H_13_O_3_);174.0330 (M-H-C_7_H_3_O_4_-2CH_3_,C_11_H_10_O_2_);146.0345(M-H-C_7_H_3_O_4_-2CH_3_-C_2_H_4_, C_9_H_6_O_2_);125.0252(M-H-C_14_H_14_O_3_,C_6_H_5_O_3_) | | | | | | | | | | | | | | | | | | |  |  |  |  |  |  |  |  |  |  |  |  |  |  |  |  |  |  |  |
| 102 | [M-H]^-^ | 33.08 | C_31_H_46_O_6_ | 513.3222 | 513.3209 | -2.5 | | | | | | 513.3221(M-H,C_31_H_45_O_6_);483.3113(M-H-CH_2_O,C_30_H_43_O_5_); 405.2894(M-H-CH_2_O-C_2_H_4_O_2_-H_2_O,C_28_H_37_O_2_) | | | | | | | | | | | | | | | Poricoic acid D | | | | | | | | | | | | | | | | | | T | | | | |
| 103 | [M+H]^+^ | 33.27 | C_20_H_18_O_5_ | 339.1227 | 339.1230 | 0.8 | | | | | | 339.1231(M+H, C_20_H_19_O_5_); 283.0608(M+H-C_4_H_8_,C_16_H_11_O_5_); 201.0546(M+H -C_6_H_6_O-CO_2_,C_13_H_13_O_2_); 137.0223(M+H-C_8_H_6_O-C_4_H_8_-CO ,C_7_H_5_O_3_) | | | | | | | | | | | | | | | | | | | | | | | | Eurycarpin A | | | | | | | | | F | | | | |
|  | [M-H]^-^ | 33.56 | C_20_H_18_O_5_ | 337.1082 | 337.1081 | 0.4 | | | | | | 337.1090(M-H,C_20_H_17_O_5_);293.0462(M-H-CO-O,C_19_H_17_O_3_);268.0377(M-H-C_5_H_9_, C_15_H_8_O_5_);224.0480(M-H-C_5_H_9_-CO-O,C_14_H_8_O_3_);135.0091(M-H-C_5_H_9_-C_8_H_5_O_2_, C_7_H_3_O_3_);117.0346(M-H-C_5_H_9_-C_7_H_2_O_3_-OH,C_8_H_5_O) | | | | | | | | | | | | | | | | | | | | | | | |  |  |  |  |  |  |  |  |  |  |  |  |  |  |
| 104 | [M+H]^+^ | 33.31 | C_21_H_20_O_6_ | 369.1333 | 369.1334 | 0.5 | | | | | | 369.1326(M+H, C_21_H_21_O_6_); 313.0698(M+H-C_4_H_8_, C_17_H_13_O_6_ ); 285.0757 (M+H-C_4_H_8_-CO, C_16_H_13_O_5_); 270.0520(M+H-C_4_H_8_-CO-CH_3_, C_15_H_10_O_5_); 185.0573(M+H-C_6_H_6_O_2_-CO_2_-2CH_3_, C_12_H_9_O_2_) | | | | | | | | | | | | | | | | | | | | | | | | Glycycoumarin | | | | | | | | | P | | | | |
|  | [M-H]^-^ | 33.59 | C_21_H_20_O_6_ | 367.1187 | 367.1187 | 0 | | | | | | 367.1193 (M-H, C_21_H_19_O_6_);337.0719(M-H-2CH_3_, C_19_H_13_O_6_);309.0414(M-H-2CH_3_ -CO,C_18_H_13_O_5_);297.0408(M-H-C_5_H_10_,C_16_H_9_O_6_); 284.0336(M-H-2CH_3_-CO-C_2_H, C_16_H_12_O_5_);203.0718 (M-H-C_6_H_5_O_2_-C_4_H_7_, C_11_H_7_O_4_) ; | | | | | | | | | | | | | | | | | | | | | | | |  |  |  |  |  |  |  |  |  |  |  |  |  |  |
| 105 | [M-H]^-^ | 33.67 | C_31_H_50_O_5_ | 501.3586 | 501.3574 | -1.1 | | | | | | 501.3209(M-H,C_31_H_49_O_5_);457.3491(M-H-CO_2_, C_30_H_49_O_3_); 439.6816(M-H-CO_2_-H_2_O,C_30_H_47_O_2_) | | | | | | | | | | 25-Hydroxy-3-epitumulosic acid | | | | | | | | | | | | | | | | | | | | | | | T | | | | |
| 106 | [M-H]^-^ | 34.18 | C_31_H_48_O_5_ | 499.3429 | 499.3414 | -3.0 | | | | | | 499.3403(M-H,C_31_H_47_O_5_);455.3562(M-H-CO_2_, C_30_H_47_O_3_) | | | | | | | | | | Poricoic acid GM | | | | | | | | | | | | | | | | | | | | | | | T | | | | |
| 107 | [M-H]^-^ | 34.75 | C_30_H_46_O_5_ | 485.3273 | 485.3261 | -1.2 | | | | | | 485.3284(M-H,C_30_H_45_O_5_);387.2550(M-H-C_6_H_10_-O,C_24_H_35_O_4_);369.2447(M-H -C_6_H_10_-O-H_2_O,C_24_H_33_O_3_);325.2159(M-H-C_6_H_10_-O-H_2_O-CO_2_,C_23_H_33_O) | | | | | | | | | | Poricoic acid G | | | | | | | | | | | | | | | | | | | | | | | T | | | | |
| 108 | [M-H]^-^ | 35.10 | C_32_H_48_O_7_ | 543.3327 | 543.3311 | -2.9 | | | | | | 543.3333(M-H,C_32_H_47_O_7_);445.2609(M-H-C_6_H_10_O,C_26_H_37_O_6_); 427.2510(M-H-C_6_H_10_O-H_2_O,C_26_H_35_O_5_) | | | | | | | | | | 26-Hydroxyporicoic acid DM | | | | | | | | | | | | | | | | | | | | | | | T | | | | |
| 109 | [M+H]^+^ | 35.27 | C_21_H_22_O_4_ | 339.1591 | 339.1592 | 0.4 | | | | | | 339.1586(M+H, C_21_H_23_O_4_); 297.1477(M+H-CO-CH_2_, C_19_H_21_O_3_ ); 245.1172(M+H-C_6_H_6_O, C_15_H_17_O_3_);177.0536(M+H-C_7_H_6_O_2_-C_2_H_2_-CH_2_, C_11_H_13_O_2_); 121.0282(M+H-C_14_H_18_O_2_, C_7_H_5_O_2_ ) | | | | | | | | | | | | | | | | | | | | | | Licochalcone A^*^ | | | | | | | | | | | F | | | | |
|  | [M-H]^-^ | 35.53 | C_21_H_22_O_4_ | 337.1445 | 337.1446 | 0.3 | | | | | | 337.145(M-H,C_21_H_21_O_4_);305.1174(M-H-CH_4_O,C_20_H_17_O_3_);229.0873(M-H-C_6_H_5_O-CH_3_,C_14_H_13_O_3_);201.0923(M-H-C_6_H_5_O-CH_3_-CO,C_13_H_13_O_2_);187.0761(M-H  -C_7_H_3_O_2_-CH_3_O,C_13_H_15_O);161.0613(M-H-C_7_H_3_O_2_-CH_3_O-C_2_H_2_,C_11_H_13_O) | | | | | | | | | | | | | | | | | | | | | |  |  |  |  |  |  |  |  |  |  |  |  |  |  |  |  |
| 110 | [M+H]^+^ | 35.31 | C_22_H_22_O_6_ | 383.1489 | 383.1489 | -0.1 | | | | | | 327.0870(M+H-C_4_H_8_, C_18_H_15_O_6_);299.0917(M+H-C_4_H_8_-CO, C_17_H_15_O_5_) ; 137.0234(M+H-C_11_H_10_O_3_, C_7_H_5_O_3_) | | | | | | | | | | | | | | | | | | | | | | | | | | | | | | | | Licoricone | F | | | | |
|  | [M-H]^-^ | 35.58 | C_22_H_22_O_6_ | 381.1344 | 381.1340 | -0.9 | | | 381.1349(M-H,C_22_H_21_O_6_);351.0876(M-H-2CH_3_,C_20_H_15_O_6_);323.0581(M-H-2CH_3_-CO, C_19_H_15_O_5_);307.0966(M-H-2CH_3_-CO-O,C_19_H_15_O_4_);279.0303(M-H-CH_3_O-C_5_H_11_,C_16_H_7_O_5_) | | | | | | | | | | | | | | | | | | | | | | | | | | | | | | | | | | |  |  |  |  |  |  |
| 111 | [M-H]^-^ | 35.39 | C_31_H_46_O_6_ | 513.3222 | 513.3205 | -3.2 | | | | | | 513.3200(M-H,C_31_H_45_O_6_);481.3324(M-H-O_2_,C_31_H_45_O_4_); 466.3098(M-H-O_2_-CH_3_,C_30_H_42_O_4_) | | | | | | | | | | | | | | | | | | Peroxydehydrotumulosic acid | | | | | | | | | | | | | | | T | | | | |
| 112 | [M-H]^-^ | 35.51 | C_20_H_20_O_5_ | 339.1238 | 339.1241 | 0.9 | | | | | | 339.1510(M-H,C_20_H_19_O_5_);187.1134(M-H-CH_3_-CO-C_6_H_5_O_2_,C_12_H_11_O_2_);132.0588(M-H-CH_3_-CO-C_6_H_5_O_2_-C_4_H_7_,C_8_H_4_O_2_);151.0042(M-H-C_8_H_5_O_2_-C_4_H_7_,C_8_H_7_O_3_) | | | | | | | | | | | | | | | | | | | | | | | | Corylifol B | | | | | | | | | F | | | | |
| 113 | [M+H]^+^ | 35.73 | C_21_H_18_O_6_ | 367.1176 | 367.1177 | 0.3 | | | | | | 367.1180(M+H,C_21_H_19_O_6_);311.0552(M+H-C_4_H_8_, C_17_H_11_O_6_); 297.0387(M+H-C_4_H_8_-CH_2_, C_16_H_9_O_6_);296.0310(M+H-C_4_H_8_-CH_3_, C_16_H_8_O_6_);253.0484(M+H-C_4_H_8_-CH_2_-CO_2_, C_15_H_9_O_4_) | | | | | | | | | | | | | | | | | | | | | | | | | | | | Glycyrol | | | | | P | | | | |
| 114 | [M-H]^-^ | 35.99 | C_20_H_18_O_5_ | 337.1082 | 337.1082 | 0 | | | | | | 337.1092(M-H,C_20_H_17_O_5_);293.0471(M-H-CO-O,C_19_H_17_O_3_); 281.0464(M-H-C_4_H_8_,C_16_H_9_O_5_) | | | | | | | | | | | | | | | | | | | | | | | | Licoflavone C | | | | | | | | | F | | | | |
|  | [M+H]^+^ | 36.31 | C_20_H_18_O_5_ | 339.1227 | 339.1223 | -1.2 | | | | | | 339.1323(M+H, C_20_H_19_O_5_); 283.0590(M+H-C_4_H_8_,C_16_H_11_O_5_); 271.0582(M+H-C_5_H_8_,C_15_H_11_O_5_); 255.0640(M+H-C_4_H_8_-CO,C_15_H_11_O_4_) | | | | | | | | | | | | | | | | | | | | | | | |  |  |  |  |  |  |  |  |  |  |  |  |  |  |
| 115 | [M+H]^+^ | 36.17 | C_20_H_16_O_5_ | 337.1071 | 337.1074 | 0.7 | | | | | | 337.1073(M+H, C_20_H_17_O_5_) ;295.0610 (M+H-C_3_H_6_, C_17_H_11_O_5_); 201.0548(M+H -C_7_H_4_O_3_,C_13_H_13_O_2_); 137.0232(M+H-C_13_H_12_O_2_, C_7_H_5_O_3_) | | | | | | | | | | | | | | | | | | | | | | | | Glabrone | | | | | | | | | F | | | | |
|  | [M-H]^-^ | 36.43 | C_20_H_16_O_5_ | 335.0925 | 335.0929 | 0.7 | | | | | | 335.0940(M-H,C_20_H_15_O_5_);320.0697(M-H-CH_3_,C_19_H_12_O_5_);319.0623(M-H-O, C_20_H_15_O_4_);305.0460(M-H-2CH_3_,C_18_H_9_O_5_);307.0981(M-H-CO,C_19_H_15_O_4_); 291.1035(M-H-O-CO,C_19_H_15_O_3_);261.0578(M-H-O-CO-2CH_3_,C_17_H_9_O_3_); 199.0768(M-H-C_7_H_4_O_3_,C_13_H_11_O_2_);183.0457(M-H-C_7_H_4_O_3_-O,C_13_H_11_O); 135.0091(M-H-C_13_H_12_O_2_,C_7_H_3_O_3_);107.0503(M-H-C_13_H_12_O_2_-CO,C_6_H_3_O_2_) | | | | | | | | | | | | | | | | | | | | | | | |  |  |  |  |  |  |  |  |  |  |  |  |  |  |
| 116 | [M+H]^+^ | 36.90 | C_15_H_20_O_2_ | 233.1536 | 233.1538 | 0.7 | | | | | | 233.1539(M+H, C_15_H_21_O_2_); 215.1430(M+H-H_2_O, C_15_H_19_O); 187.1478(M+H-H_2_O-CO, C_14_H_19_); 131.0850(M+H-H_2_O-CO-C_4_H_8_, C_10_H_11_); 105.0695 (M+H-H_2_O-CO-C_6_H_10_, C_8_H_9_) | | | | | | | | | | | | | | | | | | | | | | | | Atractylenolide Ⅱ^*^ | | | | | | | | | P | | | | |
| 117 | [M-H]^-^ | 37.08 | C_20_H_20_O_5_ | 339.1238 | 339.1235 | -0.9 | | | | | | 339.1991(M-H,C_20_H_19_O_5_);201.0956(M-H-CH_3_O-C_6_H_3_O_2_,C_13_H_13_O_2_); 183.0130(M-H-CH_3_O-O-C_6_H_5_O_2_,C_13_H_11_O) | | | | | | | | | | | | | | | | | | | | | | | | Licocoumarone | | | | | | | | | P | | | | |
| 118 | [M+H]^+^ | 37.35 | C_20_H_20_O_4_ | 325.1434 | 325.1433 | -0.6 | | | | | | 325.1448(M+H, C_20_H_21_O_4_); 137.0236(M+H-C_8_H_8_-C_4_H_8_-CO, C_7_H_5_O_3_); 103.0537(M+H-C_12_H_14_O_4_, C_8_H_7_) | | | | | | | | | | | | | | | | | | | | | | | | Glabranin | | | | | | | | | F | | | | |
|  | [M-H]^-^ | 37.59 | C_20_H_20_O_4_ | 323.1289 | 323.1286 | -0.9 | | | | | | 323.1242(M-H,C_20_H_19_O_4_);187.1129(M-H-C_6_H_5_O-CO-CH_3_,C_12_H_11_O_2_); 135.0064(M-H-C_8_H_7_O-C_5_H_9_,C_7_H_3_O_3_); 132.0582(M-H-C_6_H_5_O-CO-CH_3_-C_4_H_7_,C_8_H_4_O_2_) | | | | | | | | | | | | | | | | | | | | | | | |  |  |  |  |  |  |  |  |  |  |  |  |  |  |
| 119 | [M+H]^+^ | 37.53 | C_20_H_16_O_6_ | 353.1020 | 353.1021 | 0.5 | | | | | | 353.1022 (M+H, C_20_H_17_O_6_); 311.0544(M+H-C_3_H_6_, C_17_H_11_O_6_ );  217.0487(M+H-C_7_H_4_O_3_, C_13_H_13_O_3_); 153.0180(M+H-C_13_H_12_O_2_, C_7_H_5_O_4_) | | | | | | | | | | | | | | | | | | | | | | | | Semilicoisoflavone B | | | | | | | | | F | | | | |
| 120 | [M-H]^-^ | 37.78 | C_20_H_16_O_6_ | 351.0874 | 351.0877 | 0.7 | | | | | | 351.0898(M-H,C_20_H_15_O_6_);283.0998(M-H-C_5_H_8_,C_15_H_7_O_6_); 241.0888(M-H-C_5_H_6_O-CO,C_14_H_9_O_4_);199.0774(M-H-C_7_H_4_O_4_,C_13_H_11_O_2_) | | | | | | | | | | | | | | | | | | | | | | | | Licoisoflavone B | | | | | | | | | F | | | | |
| 121 | [M-H]^-^ | 38.09 | C_32_H_48_O_6_ | 527.3378 | 527.3371 | -1.4 | | | | | | 527.2608(M-H,C_32_H_47_O_6_);485.3285(M-H-C_3_H_6_,C_29_H_41_O_6_);467.3184(M-H -C_3_H_6_-H_2_O,C_29_H_39_O_5_);423.3280(M-H-C_3_H_6_-H_2_O-CO_2_,C_28_H_39_O_3_); 408.2983(M-H-C_3_H_6_-H_2_O-CO2-CH_3_,C_27_H_36_O_3_) | | | | | | | | | | | | | | | | | | | | | | | | Poricoic acid DM | | | | | | | | | T | | | | |
| 122 | [M+H]^+^ | 38.21 | C_22_H_22_O_6_ | 383.1489 | 383.1491 | 0.4 | | | | | | 383.1495(M+H, C_22_H_23_O_6_);299.0917(M+H-C_4_H_8_-CO, C_17_H_15_O_5_) ; 284.0660(M+H-C_4_H_8_-CO-CH_3_, C_16_H_12_O_5_) | | | | | | | | | | | | | | | | | | | | | | | | Glycyrin | | | | | | | | | P | | | | |
| 123 | [M+H]^+^ | 38.59 | C_20_H_16_O_5_ | 337.1071 | 337.1074 | 0.9 | | | | | | 337.1083(M+H, C_20_H_17_O_5_) ;295.0599 (M+H-C_3_H_6_, C_17_H_11_O_5_); 153.0182(M+H-C_13_H_12_O, C_7_H_5_O_4_) | | | | | | | | | | | | | | | | | | | | | | | | Isoderrone | | | | | | | | | F | | | | |
| 124 | [M+H]^+^ | 38.79 | C_25_H_26_O_4_ | 391.1904 | 391.1907 | 0.9 | | | | | | 391.1918(M+H, C_25_H_27_O_4_); 335.1288(M+H-C_4_H_8_, C_21_H_19_O_4_); 279.0656(M+H-2C_4_H_8_,C_17_H_11_O_4_);149.0229(M+H-C_13_H_14_O-C_4_H_8_, C_8_H_5_O_3_) | | | | | | | | | | | | | | | | | | | | | Licoflavone B | | | | | | | | | | | | F | | | | |
| 125 | [M+H]^+^ | 39.18 | C_25_H_28_O_4_ | 393.2060 | 393.2063 | 0.6 | | | | | | 393.2057(M+H, C_25_H_29_O_4_); 337.1436(M+H-C_4_H_8_, C_21_H_21_O_4_); 149.0234(M+H-C_4_H_8_-C_13_H_16_O, C_8_H_5_O_3_) | | | | | | | | | | | | | | | | | | | | | | | | Glabrol | | | | | | | | | F | | | | |
|  | [M-H]^-^ | 39.40 | C_25_H_28_O_4_ | 391.1915 | 391.1917 | 0.5 | | | | | | 391.1916(M-H,C_25_H_27_O_4_);203.0720(M-H-C_13_H_16_O, C_12_H_11_O_3_);187.1137 (M-H-C_12_H_12_O_3_, C_13_H_15_O);132.0584(M-H-C_12_H_12_O_3_-C_4_H_7_, C_9_H_8_O) | | | | | | | | | | | | | | | | | | | | | | | |  |  |  |  |  |  |  |  |  |  |  |  |  |  |
| 126 | [M+H]^+^ | 39.75 | C_22_H_26_O_5_ | 371.1853 | 371.1856 | 0.8 | | | | | | 181.0860(M+H-C_8_H_6_O_2_-C_4_H_8_,C_10_H_13_O_3_); 167.0700 (M+H-C_9_H_8_O_2_ -C_4_H_8_,C_9_H_11_O_3_); 137.0589(M+H-C_14_H_18_O_3_,C_8_H_9_O_2_); 123.0441(M+H-C_9_H_10_O_2_-C_5_H_10_-2CH_2_,C_6_H_3_O_3_) | | | | | | | | | | | | | | | | | | | | | | | | Glyasperin D | | | | | | | | | F | | | | |
|  | [M-H]^-^ | 39.98 | C_22_H_26_O_5_ | 369.1707 | 369.1707 | 0 | | | | | | 369.1725(M-H,C_22_H_25_O_5_) ; 339.1247 (M-H-2CH_3_,C_20_H_19_O_5_); 221.1183(M-H-C_9_H_8_O_2_,C_13_H_17_O_3_); 203.0728(M-H-C_6_H_6_O_2_-C_4_H_8_, C_12_H_11_O_3_); 135.0453(M-H-C_14_H_18_O_3_, C_8_H_7_O_2_) ; 109.0925(M-H-C_14_H_18_O_3_-C_2_H_2_, C_6_H_5_O_2_) | | | | | | | | | | | | | | | | | | | | | | | |  |  |  |  |  |  |  |  |  |  |  |  |  |  |
| 127 | [M+H]^+^ | 39.95 | C_15_H_18_O_2_ | 231.1380 | 231.1381 | 0.9 | | | | | | 231.1381(M+H, C_15_H_19_O_2_); 203.1087(M+H-CO, C_14_H_19_O); 185.1325(M+H-2CH_3_-O, C_13_H_13_O); 157.1011(M+H-2CH_3_-O-CO, C_12_H_13_); 143.0846(M+H-C_3_H_8_-CO_2_, C_11_H_11_); 128.0615(M+H-C_4_H_8_O_2_-CH_3_,C_10_H_8_) | | | | | | | | | | | | | | | | | | | | | | | | Atractylenolide I^*^ | | | | | | | | | L | | | | |
| 128 | [M-H]^-^ | 40.64 | C_30_H_44_O_5_ | 483.3116 | 483.3105 | -2.2 | | | | | | 483.3079(M-H,C_30_H_43_O_5_);409.2729(M-H-C_3_H_6_O_2_,C_27_H_37_O_3_) | | | | | | | | | | | | Poricoic acid B^*^ | | | | | | | | | | | | | | | | | | | | | T | | | | |
| 129 | [M-H]^-^ | 40.84 | C_31_H_48_O_4_ | 483.3480 | 483.3471 | -1.8 | | | | | | 483.3483(M-H,C_31_H_47_O_4_);439.3550(M-H-CO_2_,C_30_H_47_O_2_); 313.2218(M-H-C_9_H_15_O_2_-CH_3_,C_21_H_29_O_2_) | | | | | | | | Dehydrotumulosic acid^*^ | | | | | | | | | | | | | | | | | | | | | | | | | T | | | | |
| 130 | [M-H]^-^ | 41.35 | C_31_H_50_O_4_ | 485.3636 | 485.3626 | -0.2 | | | | | | 485.3639(M-H,C_31_H_49_O_4_);423.3196(M-H-H_2_O-CO_2_,C_30_H_47_O) | | | | | | | | | | | | | | | | | | | | Tumulosic acid | | | | | | | | | | | | | T | | | | |
| 131 | [M+H]^+^ | 41.83 | C_25_H_26_O_6_ | 423.1802 | 423.1802 | -0.1 | | | | | | 423.1807(M+H,C_25_H_27_O_6_);367.1182 (M+H-C_4_H_8_,C_21_H_19_O_6_);  311.0550 (M+H-C_5_H_8_-CO_2_, C_19_H_19_O_4_) | | | | | | | | | | | | | | | | | | | | Sanggenol L | | | | | | | | | | | | | F | | | | |
| 132 | [M-H]^-^ | 42.04 | C_25_H_26_O_6_ | 421.1657 | 421.1656 | -0.2 | | | | | | 421.1663(M-H,C_25_H_25_O_6_);377.1038(M-H-CO-O,C_24_H_25_O_4_);365.1027(M-H-C_4_H_8_,C_21_H_17_O_6_);352.0956(M-H-C_5_H_9_,C_20_H_16_O_6_);309.0404(M-H-2C_4_H_8_,C_17_H_9_O_6_) | | | | | | | | | | | | | | | | | | | | Isoangustone A | | | | | | | | | | | | | F | | | | |
| 133 | [M-H]^-^ | 42.09 | C_31_H_46_O_5_ | 497.3272 | 497.3261 | -2.2 | | | | | | 497.3272(M-H,C_31_H_45_O_5_);424.2994(M-H-C_3_H_5_O_2_,C_28_H_40_O_3_);423.2922(M-H -C_3_H_5_O_2_-H,C_28_H_39_O_3_);379.2940(M-H-C_3_H_5_O_2_-H-CO_2_,C_27_H_39_O) | | | | | | | | | | | | | | | | | | | | Poricoic acid A^*^ | | | | | | | | | | | | | T | | | | |
| 134 | [M-H]^-^ | 42.09 | C_32_H_48_O_6_ | 527.3378 | 527.3363 | -2.8 | | | | | | 527.3402(M-H,C_32_H_47_O_6_);481.2919(M-H-CH_2_O_2_,C_31_H_45_O_4_);429.2676 (M-H-C_6_H_10_O,C_26_H_37_O_5_);411.2540(M-H-C_6_H_10_O-H_2_O,C_26_H_35_O_4_) | | | | | | | | | | | | | | | | | | | | 25-Methoxyporicoic acid A | | | | | | | | | | | | | T | | | | |
| 135 | [M+H]^+^ | 42.49 | C_21_H_20_O_5_ | 353.1384 | 353.1385 | 0.6 | | | | | | 353.1409(M+H, C_21_H_21_O_5_);297.0764(M+H-C_4_H_8_, C_17_H_13_O_5_); 267.0642 (M+H-C_4_H_8_-CH_2_O, C_16_H_11_O_4_); 239.0698(M+H-C_4_H_8_-CH_2_O-CO, C_15_H_11_O_3_); 149.0219(M+H-C_12_H_12_O_3_, C_9_H_9_O_2_); 121.0283(M+H-C_12_H_12_O_3_-CO, C_8_H_9_O) | | | | | | | | | | | | | | | | | | | | Gancanin M | | | | | | | | | | | | | F | | | | |
| 136 | [M-H]^-^ | 42.51 | C_30_H_46_O_4_ | 469.3324 | 469.3313 | -2 | | | | | | 469.3324(M-H,C_30_H_45_O_4_);425.3417(M-H-CO_2_,C_29_H_45_O_2_) | | | | | | | | | 16α-Hydroxydehydrotrametenolic acid | | | | | | | | | | | | | | | | | | | | | | | | | | | T | |
| 137 | [M-H]^-^ | 42.51 | C_30_H_46_O_4_ | 469.3323 | 469.3313 | -2.1 | | | | | | 469.3324(M-H, C_30_H_45_O_4_);425.3417(M+H-CO_2_,C_29_H_45_O_2_ ) | | | | | | Glycyrrhetinic acid^*^ | | | | | | | | | | | | | | | | | | | | | | | | | | | T | | | | |
| 138 | [M-H]^-^ | 42.75 | C_33_H_52_O_6_ | 543.3691 | 543.3678 | -2.5 | | | | | | 543.3699(M-H,C_33_H_51_O_6_);467.3575(M-H-C_2_H_3_O-H_2_O-CH_3_,C_30_H_43_O_4_) | | | | | 25-Hydroxypachymic acid | | | | | | | | | | | | | | | | | | | | | | | | | | | | T | | | | |
| 139 | [M-H]^-^ | 42.90 | C_31_H_46_O_4_ | 481.3323 | 481.3312 | -1.7 | | | | | | 481.3320(M-H,C_31_H_45_O_4_);421.3029(M-H-C_2_H_4_O_2_, C_29_H_41_O_2_) | | | | | | Polyporenic acid C^*^ | | | | | | | | | | | | | | | | | | | | | | | | | | | T | | | | |
| 140 | [M+H]^+^ | 43.64 | C_25_H_26_O_5_ | 407.1853 | 407.1852 | -0.4 | | | | | | 407.2925(M+H, C_25_H_27_O_5_); 351.1214(M+H-C_4_H_8_, C_21_H_19_O_5_) ; 295.0601 (M+H-C_4_H_8_-C_4_H_8_, C_17_H_11_O_5_) ;177.0176(M+H-C_4_H_8_-C_4_H_8_-C_8_H_6_O, C_9_H_5_O_4_) | | | | | | | | | | | | | | | | | | | | | | | | 6,8-Diprenylgenistein | | | | | | | | | F | | | | |
|  | [M-H]^-^ | 44.75 | C_25_H_26_O_5_ | 405.1708 | 405.1704 | -1 | | | | | | 405.1703(M-H,C_25_H_25_O_5_);219.0677(M-H-C_8_H_6_O-C_5_H_8_,C_12_H_11_O_4_); 185.0979(M-H-C_5_H_9_-C_6_H_4_O-CH_3_-CO_2_,C_12_H_9_O_2_) | | | | | | | | | | | | | | | | | | | | | | | |  |  |  |  |  |  |  |  |  |  |  |  |  |  |
| 141 | [M+H]^+^ | 44.55 | C_25_H_26_O_5_ | 407.1853 | 407.1855 | -0.4 | | | | | | 407.1882(M+H,C_25_H_27_O_5_);351.1233(M+H-C_4_H_8_,C_21_H_19_O_5_ );187.1135(M+H -C_12_H_12_O_4_,C_13_H_15_O);165.0182(M+H-C_4_H_8_-C_13_H_14_O,C_8_H_5_O_4_);137.0240(M+H -C_4_H_8_-C_13_H_14_O-CO,C_7_H_5_O_3_) | | | | | | | | | | | | | | | | | | | | | | | | Isolupalbigenin | | | | | | | | | F | | | | |
| 142 | [M-H]^-^ | 44.62 | C_30_H_44_O_4_ | 467.3167 | 467.3159 | -3.5 | | | | | | 467.3192(M-H,C_30_H_43_O_4_);423.3277(M-H-CO_2_,C_29_H_43_O_2_) | | 16-Deoxyporicoic acid B | | | | | | | | | | | | | | | | | | | | | | | | | | | | | | | T | | | | |
| 143 | [M-H]^-^ | 46.73 | C_32_H_50_O_5_ | 513.3586 | 513.3568 | -3.4 | | | | | | 513.3585(M-H,C_32_H_49_O_5_);451.3157(M-H-CO_2_-H_2_O,C_31_H_47_O_2_) | | Poricoic acid HM | | | | | | | | | | | | | | | | | | | | | | | | | | | | | | | T | | | | |
| 144 | [M-H]^-^ | 47.03 | C_30_H_48_O_3_ | 455.3530 | 455.3524 | -1.5 | | | | | | 455.3528(M-H,C_30_H_47_O_3_);339.2696(M-H-CO_2_-H_2_O-C_4_H_6_, C_25_H_39_) | | Trametenolic acid | | | | | | | | | | | | | | | | | | | | | | | | | | | | | | | T | | | | |
| 145 | [M-H]^-^ | 47.39 | C_33_H_50_O_5_ | 525.3578 | 525.3578 | -1.4 | | | | | | 525.3587(M-H,C_33_H_49_O_5_);481.3641(M-H-CO_2_, C_32_H_49_O_3_) | | Dehydropachymic acid | | | | | | | | | | | | | | | | | | | | | | | | | | | | | | | T | | | | |
| 146 | [M+H]^+^ | 48.01 | C_33_H_52_O_5_ | 529.3886 | 529.3890 | 0.2 | 511.3770(M+H-H_2_O,C_33_H_51_O_4_);451.3562(M+H-H_2_O-C_2_H_4_O_2_, C_31_H_47_O_2_); 355.2625 (M+H-H_2_O-C_2_H_4_O_2_-C_7_H_12_, C_24_H_35_O_2_); 295.2422(M+H-H_2_O-C_2_H_4_O_2_-C_7_H_12_-C_2_H_4_O_2_, C_22_H_31_) | | | | | | | | | | | | | | | | | | | | | | | | | | | | | | | | | | Pachymic acid^*^ | | | | T | | | | |
|  | [M-H]^-^ | 48.15 | C_33_H_52_O_5_ | 527.3742 | 527.3736 | -0.4 | | | | | | 527.3737(M-H,C_33_H_51_O_5_);467.3529(M-H-C_2_H_4_O_2_, C_31_H_47_O_3_); 465.3368(M-H-CO_2_-H_2_O, C_32_H_49_O_2_); 405.3179(M-H-CO_2_-H_2_O-C_2_H_4_O_2_, C_30_H_45_); 293.1899(M-H-CO_2_-H_2_O-C_2_H_4_O_2_-C_8_H_16_,C_22_H_29_) | | | | | | | | | | | | | | | | | | | | | | | | | | | | |  |  |  |  |  |  |  |  |  |
| 147 | [M-H]^-^ | 49.88 | C_16_H_30_O_2_ | 253.2173 | 253.2176 | 0.5 | | | | | | 253.2179(M-H,C_16_H_29_O_2_);235.2044(M-H-H_2_O,C_16_H_27_O) | | Sclareol Glycol | | | | | | | | | | | | | | | | | | | | | | | | | | | | | | | O | | | | |
| 148 | [M-H]^-^ | 50.65 | C_30_H_46_O_3_ | 453.3374 | 453.3365 | -3.4 | | | | | | 453.3380(M-H,C_30_H_45_O_3_) | | | 3β-Hydroxylanosta-7,9(11),24-trien-21-oic acid^*^ | | | | | | | | | | | | | | | | | | | | | | | | | | | | | | | | | T | |
| 149 | [M-H]^-^ | 52.30 | C_31_H_48_O_3_ | 467.3531 | 467.3527 | -0.8 | | | | | | 467.3516(M+H, C_31_H_47_O_3_);339.1988(M-H-C_5_H_9_-CO_2_-CH_3_, C_24_H_35_O) | | | | | | | | | | | | | | | | | | | | | | | | Dehydroeburicoic acid | | | | | | | | | T | | | | |

Note: *Identified by comparing with reference standards. F: flavonoids; T: triterpenoids; P: phenylpropanoids; OA: organic acids; AL: alkaloids; L: lactones; AL: alkaloids; AN: anthraquinones; O: other types.
